# Supplementary material for: Accurate prediction of protein folding mechanisms by simple structure-based statistical mechanical models
Source: Nat Commun. 2023 Oct 19;14:6338. doi: 10.1038/s41467-023-41664-1 (PMC10587348; doi:10.1038/s41467-023-41664-1)
Supplement: Supplementary file 1 — Supplementary Information [file 41467_2023_41664_MOESM1_ESM.pdf]

Supplementary Information

Accurate prediction of protein folding mechanisms by simple structure-based statistical mechanical models

Koji Ooka[1,2] and Munehito Arai[1,2,3,*]

[1] Department of Physics, Graduate School of Science, The University of Tokyo, 3-8-1 Komaba, Meguro, Tokyo 153-8902, Japan

[2] Komaba Organization for Educational Excellence, College of Arts and Sciences, The University of Tokyo, 3-8-1 Komaba, Meguro, Tokyo 153-8902, Japan

[3] Department of Life Sciences, Graduate School of Arts and Sciences, The University of Tokyo, 3-8-1 Komaba, Meguro, Tokyo 153-8902, Japan

*Corresponding author

Email: arai@bio.c.u-tokyo.ac.jp

Contents

Supplementary Tables 4
Supplementary Table 1. Proteins used in this study.......... 4
Supplementary Table 2. The contact energy size ε and the scaling factor of the ring entropy $h_{s'}$ used for each protein.......... 5
Supplementary Figures.......... 6
Supplementary Fig. 1. Folding of engrailed homeodomain (En-HD).......... 6
Supplementary Fig. 2. Folding of src SH3 domain.......... 8
Supplementary Fig. 3. Folding of α-spectrin SH3 domain. 9
Supplementary Fig. 4. Folding of cold shock protein B (CspB).......... 10
Supplementary Fig. 5. Folding of chymotrypsin inhibitor 2 (CI2). 11
Supplementary Fig. 6. Folding of activation domain of human procarboxypeptidase A2 (ADA2h).......... 12
Supplementary Fig. 7. Correlation between experimental results and predictions for small single-domain proteins. 13
Supplementary Fig. 8. Experimentally determined folding pathways of large multidomain proteins.......... 15
Supplementary Fig. 9. Folding of apomyoglobin (apoMb). I. 16
Supplementary Fig. 10. Folding of apomyoglobin (apoMb). II.......... 17
Supplementary Fig. 11. Folding of barnase. I.......... 18
Supplementary Fig. 12. Folding of barnase. II.......... 19
Supplementary Fig. 13. Folding of ribonuclease HI (RNase HI). I. 20
Supplementary Fig. 14. Folding of ribonuclease HI (RNase HI). II.......... 21
Supplementary Fig. 15. Folding of dihydrofolate reductase (DHFR). I. 22
Supplementary Fig. 16. Folding of dihydrofolate reductase (DHFR). II.......... 23
Supplementary Fig. 17. Folding of α-subunit of tryptophan synthase (αTS). I. 24
Supplementary Fig. 18. Folding of α-subunit of tryptophan synthase (αTS). II.......... 25
Supplementary Fig. 19. Folding of indole-3-glycerol phosphate synthase (IGPS). I. 26
Supplementary Fig. 20. Folding of indole-3-glycerol phosphate synthase (IGPS). II.......... 27
Supplementary Fig. 21. Experimentally determined protein folding pathways involving oxidative disulfide bond formation. 28
Supplementary Fig. 22. Oxidative folding of hen egg-white lysozyme. I. 29
Supplementary Fig. 23. Oxidative folding of hen egg-white lysozyme. II.......... 30
Supplementary Fig. 24. Oxidative folding of ribonuclease A (RNase A). I. 31
Supplementary Fig. 25. Oxidative folding of ribonuclease A (RNase A). II.......... 32
Supplementary Fig. 26. Oxidative folding of bovine pancreatic trypsin inhibitor (BPTI). I. 33
Supplementary Fig. 27. Oxidative folding of bovine pancreatic trypsin inhibitor (BPTI). II. 34
Supplementary Fig. 28. Experimentally determined folding pathways of disulfide-intact proteins.......... 35
Supplementary Fig. 29. Folding of disulfide-intact hen egg-white lysozyme. I.......... 36
Supplementary Fig. 30. Folding of disulfide-intact hen egg-white lysozyme. II.......... 37
Supplementary Fig. 31. Kinetic analysis of lysozyme folding at 293 K.......... 38
Supplementary Fig. 32. Kinetic analysis of lysozyme folding at 308 K.......... 39
Supplementary Fig. 33. Kinetic analysis of lysozyme folding at 323 K.......... 40
Supplementary Fig. 34. Kinetic analysis of lysozyme folding at 338 K.......... 41
Supplementary Fig. 35. Temperature dependence of free energy landscape of lysozyme. . 42
Supplementary Fig. 36. Two-dimensional free energy landscapes of lysozyme with a single disulfide bond calculated using the WSME-L(SS_{intact}) model. 43

Supplementary Fig. 37. Folding of disulfide-intact ribonuclease A (RNase A). I. 44
Supplementary Fig. 38. Folding of disulfide-intact ribonuclease A (RNase A). II. 45
Supplementary Fig. 39. Folding of disulfide-intact bovine pancreatic trypsin inhibitor (BPTI). I. 46
Supplementary Fig. 40. Folding of disulfide-intact bovine pancreatic trypsin inhibitor (BPTI). II. 47
Supplementary Fig. 41. Kinetic analysis of lysozyme folding considering non-native interactions. 48
Supplementary Fig. 42. Two-dimensional free energy landscape of lysozyme calculated by WSME-L model using uniform contact energy at 293 K. 49
Supplementary Fig. 43. Molecular dynamics simulations of apomyoglobin. 50
Supplementary Fig. 44. Folding of disulfide-intact lysozyme with simultaneous introduction of four linkers. 51
Supplementary References 52

Supplementary Tables

Supplementary Table 1. Proteins used in this study.

Protein	PDB ID	Chain length [a]	Class [b]	ΔG_{NU} (in $k_B T$ unit) [c]	References of experimental results
Small proteins					
En-HD	2jwt	59	all α	2.9	[1]
src SH3	4jz4	60	all β	6.9	[2]
α-spectrin SH3	1u06	55	all β	6.6	[3]
CspB	1csp	67	all β	6.4	[4]
CI2	7a1h	64	α+β	12.8	[5]
ADA2h	1aye	78	α+β	6.9	[6]
Large proteins					
apoMb	1bzp	153	all α	10.9	[7-9]
barnase	1a2p	108	α+β	17.2	[10-13]
RNase HI	7vsc	155	α/β	16.4	[14-16]
DHFR	5uih	159	α/β	10.3	[17-20]
αTS	AF-P0A877-F1	268	α/β	18.3	[21-23]
IGPS	1jul	223	α/β	22.1	[24]
Oxidative folding & folding of disulfide-intact proteins					
lysozyme	1iee	129	α+β	16	[25-39]
RNase A	6etl	124	α+β	15.2	[40-49]
BPTI	5pti	58	small	15.2	[50-53]

[a] The number of residues registered in the PDB file.

[b] Classification based on SCOP2[54].

[c] Stability of the protein, represented as the difference in the free energy between the native and unfolded states in water, taken from the references.

Supplementary Table 2. The contact energy size ε and the scaling factor of the ring entropy $h_{s'}$ used for each protein.

Protein	ε (kcal/mol) for Original model 1	ε (kcal/mol) for Original model 2	ε (kcal/mol) for WSME-L models	$h_{S'}$
Small proteins				
En-HD	0.3038	1.490	1.868	1.1
src SH3	0.2949	2.007	2.506	0.5
α-spectrin SH3	0.2854	1.659	2.063	1.4
CspB	0.3067	1.506	1.836	0.5
CI2	0.3375	5.806	7.177	1.9
ADA2h	0.2884	2.552	3.155	0.5
Large proteins				
apoMb	0.3088	2.439	2.779	0.5
barnase	0.2984	5.520	6.525	2.0
RNase HI	0.2712	3.668	4.507	1.5
DHFR	0.2688	2.945	3.643	0.5
αTS	0.2412	4.949	6.191	0.5
IGPS	0.2494	3.592	4.466	0.5
Oxidative folding				
lysozyme	0.2274	1.204	1.526	0.5
RNase A	0.2273	2.585	3.214	2.0
BPTI	0.2363	1.482	1.904	0.5
Folding of disulfide-intact proteins				
lysozyme	0.2690	1.431	1.783	1.0
RNase A	0.2758	2.227	2.733	2.0
BPTI	0.3446	2.334	2.788	2.0

Supplementary Figures

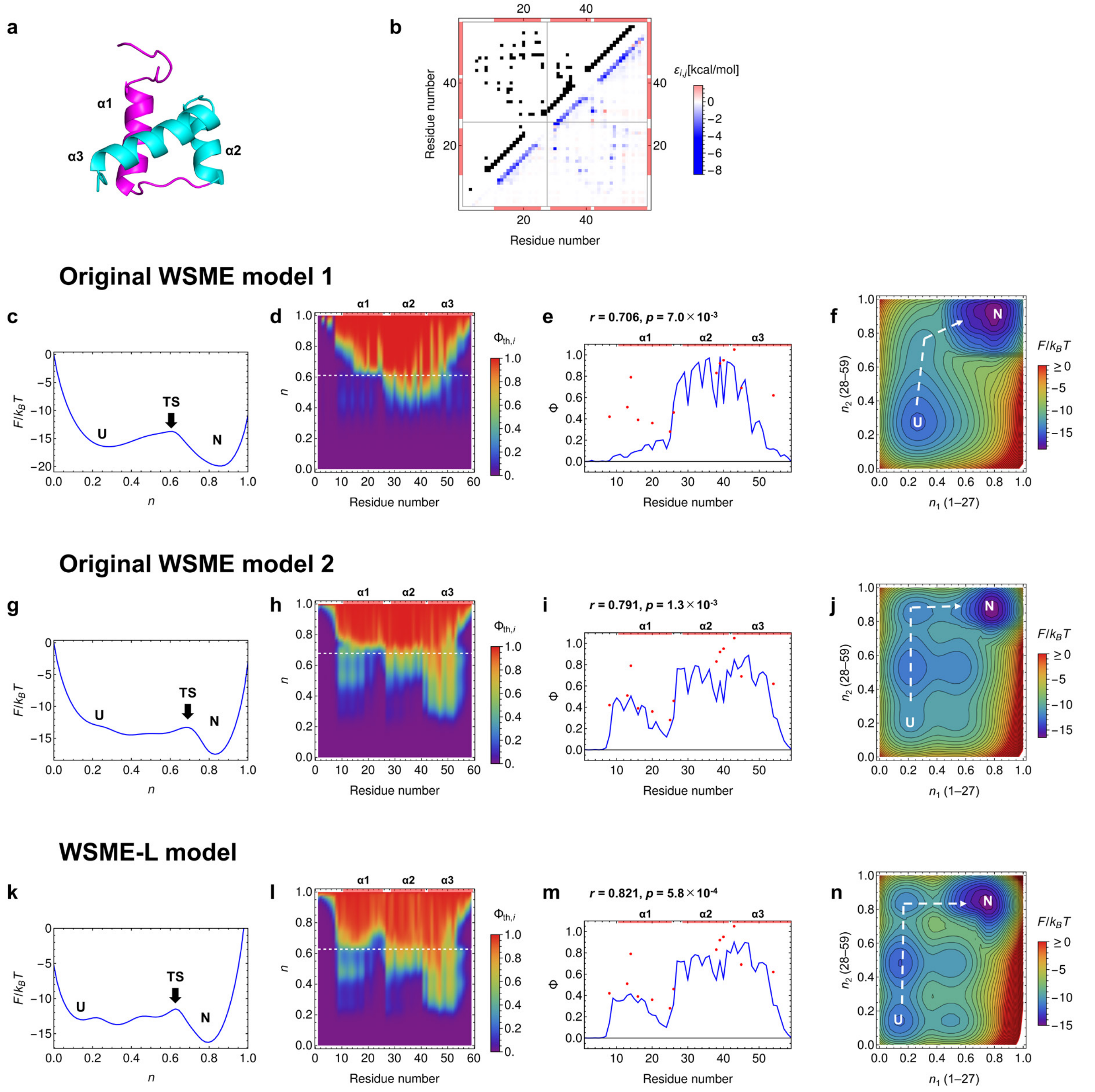

Supplementary Fig. 1. Folding of engrailed homeodomain (En-HD).
a Native structure. N-terminal (residues 1–27) and C-terminal (residues 28–59) halves are shown in magenta and cyan, respectively. **b** Contact map (top left) and AMBER-derived contact energy (bottom right). **c, g, k** One-dimensional free energy landscapes. U, TS, and N denote unfolded, transition, and native states, respectively. **d, h, l** Residue-specific structure formation predicted by theoretical Φ-value analysis. White dashed line indicates position of transition state. **e, i, m** Theoretical Φ-values in transition state (blue line) and experimental Φ-values (red filled circles). Correlation coefficients between them, *r*, and *p*-value of two-sided *t*-test without adjustments are shown. In **b**, **e**, **i**, **m**, red and green boxes at top indicate locations of helices and strands, respectively. **f, j, n** Two-dimensional (2D) free energy landscapes. n_1 and n_2 are order parameters for N- and C-terminal halves, respectively. Dominant folding pathway is indicated by white dashed line. Panels **c–f**, **g–j**, and **k–n** were predicted by Original models 1 and 2 and WSME-L model, respectively. Original model 2 predicted experimental Φ-values well (**i**), and WSME-L model further improved this prediction (**m**). In **n**, 2D free energy landscape predicted by WSME-L model suggests existence of multiple folding pathways,

consistent with experimental results showing that En-HD folds via a diffusion-collision mechanism. Source data are provided as a Source Data file.

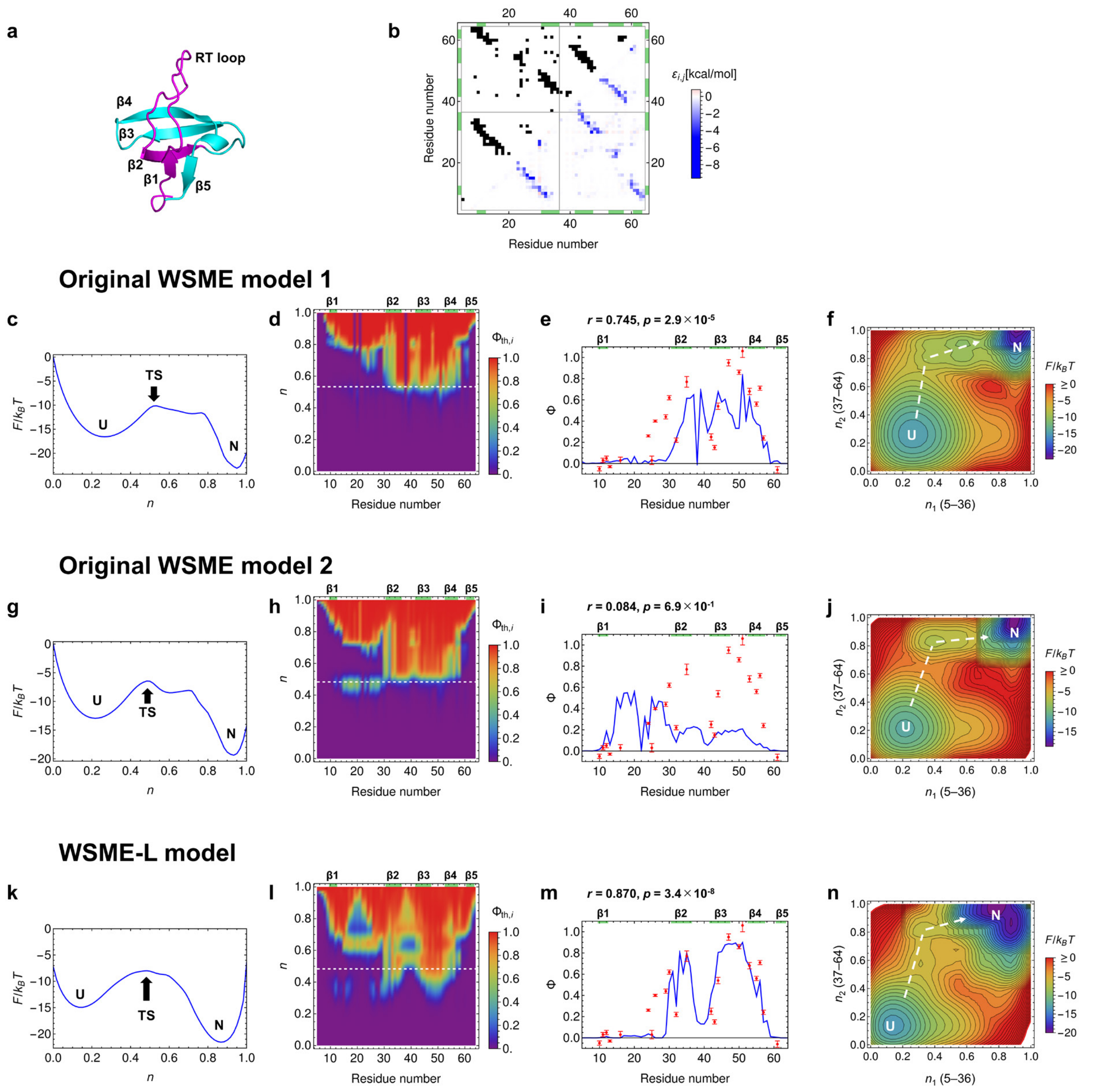

Supplementary Fig. 2. Folding of src SH3 domain.

a–n Details are shown in Supplementary Fig. 1. In **a**, N-terminal (residues 5–36) and C-terminal (residues 37–64) halves are shown in magenta and cyan, respectively. WSME-L model predicted values in agreement with experiment (**m**), while Original model 2 predicted very poor values (**i**). Source data are provided as a Source Data file.

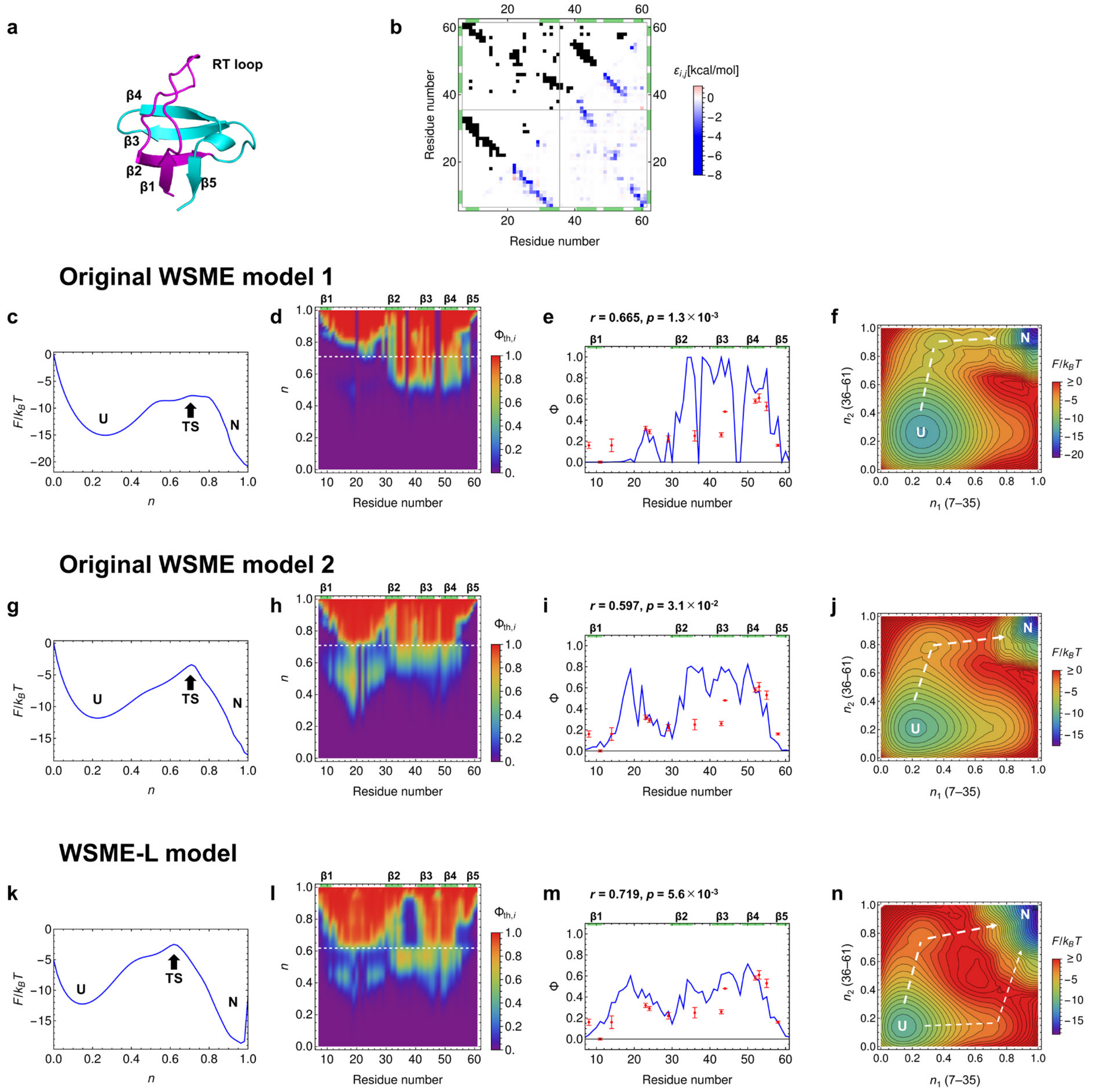

Supplementary Fig. 3. Folding of α-spectrin SH3 domain.

a–n Details are shown in Supplementary Fig. 1. In **a**, N-terminal (residues 7–35) and C-terminal (residues 36–61) halves are shown in magenta and cyan, respectively. The α-spectrin SH3 and src SH3 domains have similar structures but different folding mechanisms. Based on the two-dimensional free energy landscape predicted by WSME-L model (**n**), the α-spectrin SH3 domain was predicted to have two folding pathways: major pathway involving folding from C-terminal region, which is similar to src SH3 domain, and minor pathway involving folding from N-terminal region. Thus, WSME-L model can successfully explain differences in folding pathways of two proteins with similar structures. Source data are provided as a Source Data file.

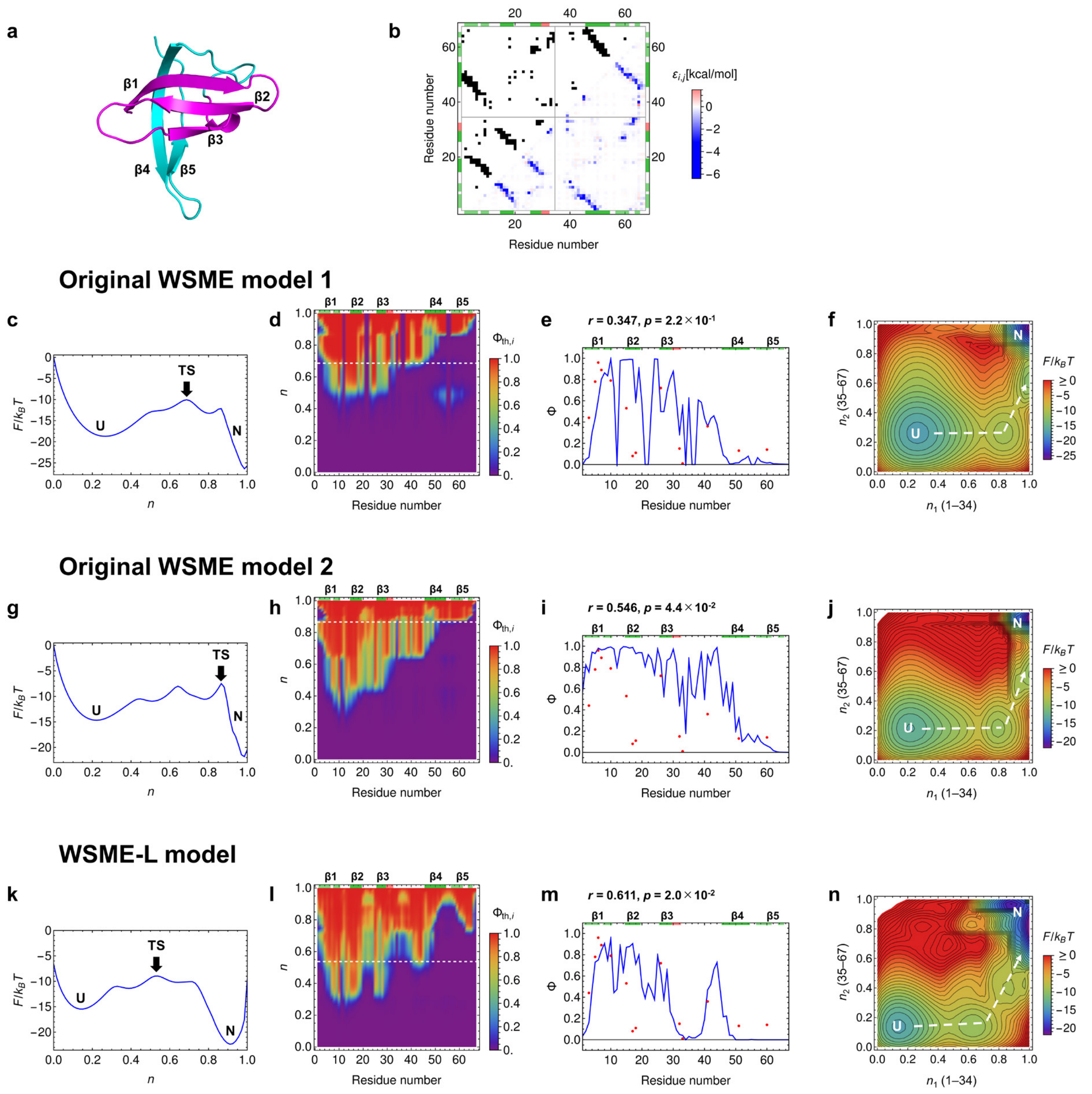

Supplementary Fig. 4. Folding of cold shock protein B (CspB).
a–n Details are shown in Supplementary Fig. 1. In **a**, N-terminal (residues 1–34) and C-terminal (residues 35–67) halves are shown in magenta and cyan, respectively. All three models predicted folding of N-terminal β-strands in transition state. In **e**, **i**, **m**, values predicted by WSME-L model were more consistent with the experimental ones, as indicated by the highest correlation coefficient. Source data are provided as a Source Data file.

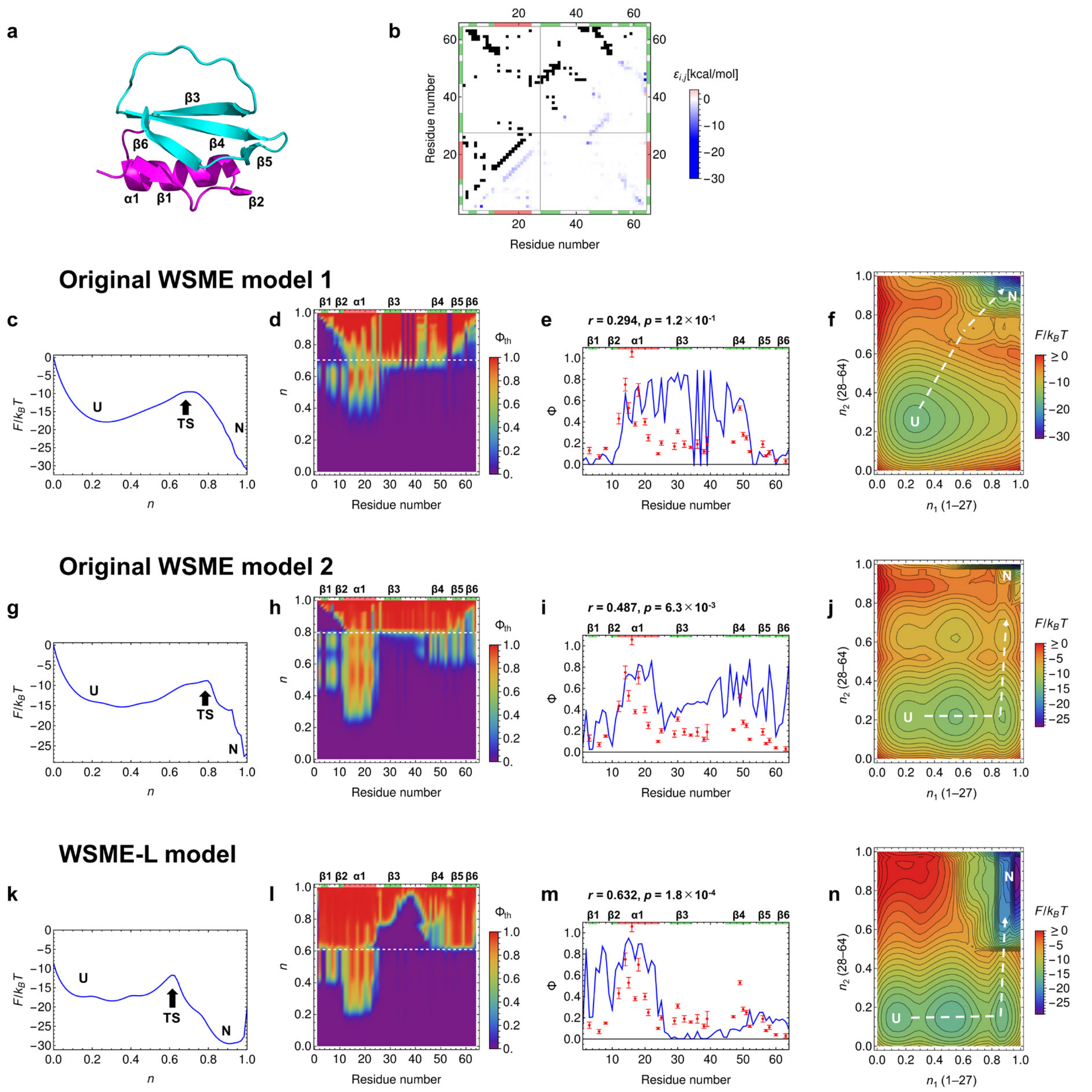

Supplementary Fig. 5. Folding of chymotrypsin inhibitor 2 (CI2).
a–n Details are shown in Supplementary Fig. 1. In **a**, N-terminal (residues 1–27) and C-terminal (residues 28–64) halves are shown in magenta and cyan, respectively. Original model 1 failed to predict values consistent with experimental ones (**e**), but WSME-L model exhibited improved prediction (**m**). Source data are provided as a Source Data file.

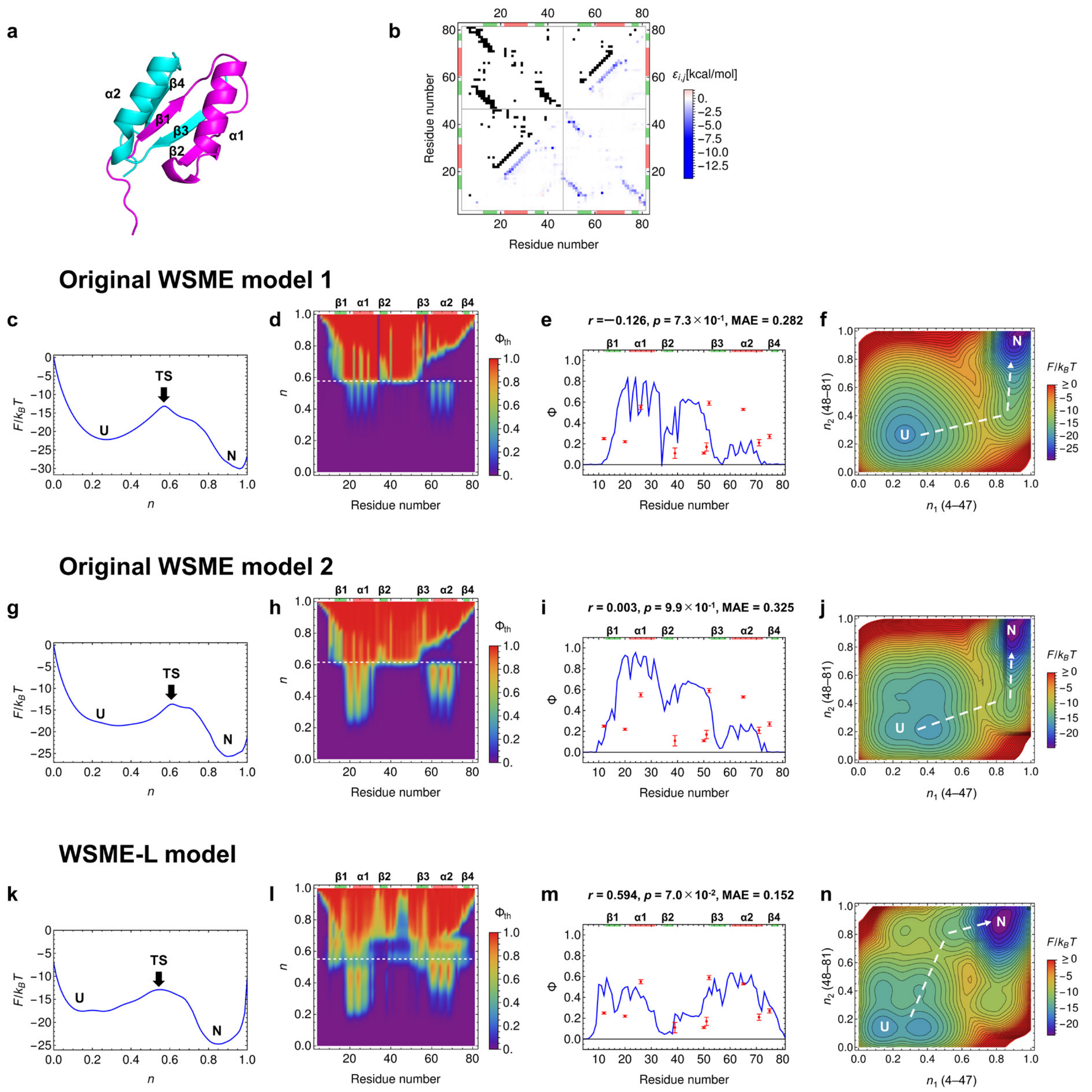

Supplementary Fig. 6. Folding of activation domain of human procarboxypeptidase A2 (ADA2h).

a–n Details are shown in Supplementary Fig. 1. In **a**, N-terminal (residues 4–47) and C-terminal (residues 48–81) halves are shown in magenta and cyan, respectively. Original models failed to predict values consistent with experimental results (**e**, **i**). In contrast, WSME-L model predicted folding pathway different from that of original models (**n**) and improved the prediction of Φ-values (**m**). Source data are provided as a Source Data file.

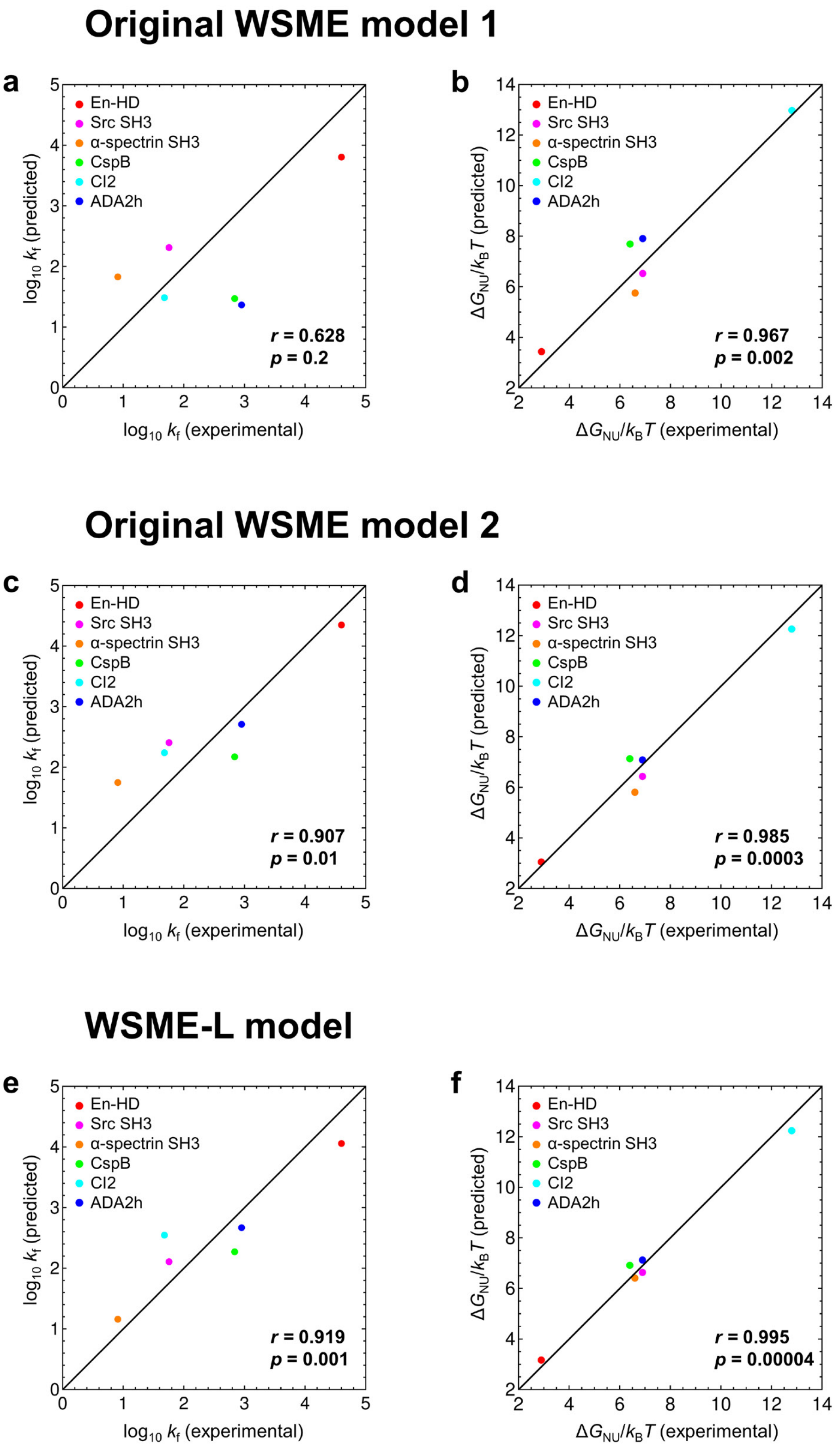

Supplementary Fig. 7. Correlation between experimental results and predictions for small single-domain proteins.

a, c, e Correlation between experimentally measured folding rates and those predicted by Original model 1 (**a**), Original model 2 (**c**), and WSME-L model (**e**). **b, d, f** Correlation between experimentally measured stabilities and those predicted by Original model 1 (**b**), Original model 2 (**d**), and WSME-L model (**f**). Correlation coefficients, *r*, and *p*-values of two-sided *t*-test without adjustments are shown. Source data are provided as a Source Data file.

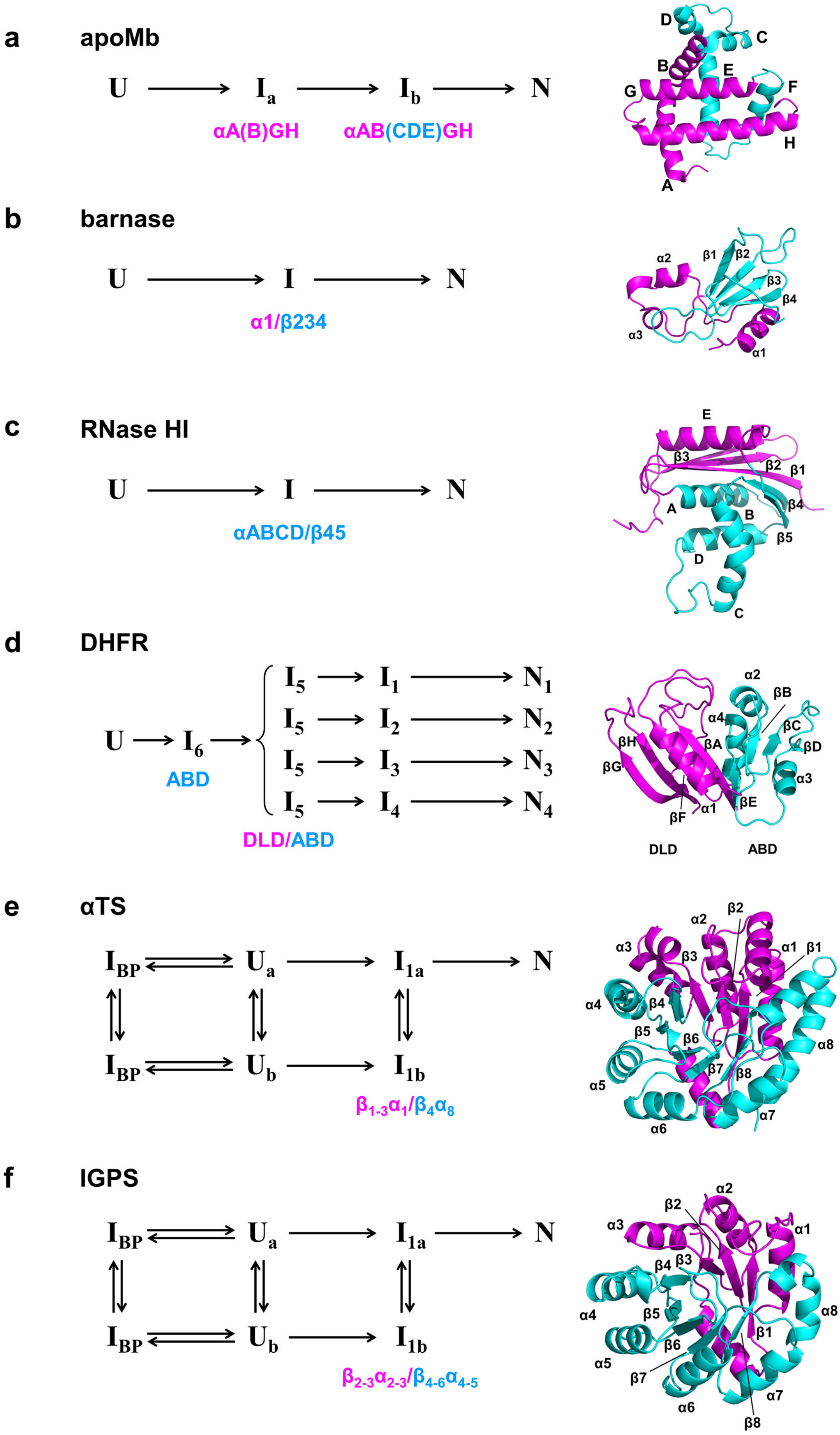
a
apoMb
U → I_a → I_b → N
αA(B)GH
αAB(CDE)GH
D
C
B
E
G
F
H
A
b
barnase
U → I → N
α1/β234
α2
β1
β2
β3
β4
α3
α1
c
RNase HI
U → I → N
αABCD/β45
E
β3
β2
β1
A
B
β4
β5
D
C
d
DHFR
U → I6 → I5 → I1 → N1
I5 → I2 → N2
I5 → I3 → N3
I5 → I4 → N4
ABD
DLD/ABD
α2
βB
α4
βC
βH
βA
βD
βG
α3
α1
βE
βF
DLD
ABD
e
αTS
IBP ⇄ Ua → I1a → N
IBP ⇄ Ub → I1b
β1-3α1/β4α8
α2
β2
α3
β3
α1
β1
α4
β4
β5
β6
α8
β7
β8
α5
α7
α6
f
IGPS
IBP ⇄ Ua → I1a → N
IBP ⇄ Ub → I1b
β2-3α2-3/β4-6α4-5
α2
β2
α3
α1
β4
β3
α4
β5
α8
β1
β6
α5
β7
α7
α6
β8

Supplementary Fig. 8. Experimentally determined folding pathways of large multidomain proteins.

a Folding of apomyoglobin (apoMb). The A, (B), G, and H helices are formed in the early folding intermediate, whereas the C, D, and E helices are formed late[8,9]. The F helix is not stably folded in the native state of apoMb[7]. U and N denote the unfolded and native states, respectively. **b** Folding of barnase. Both helix 1 (residues 7–17) and strands 2–4 (residues 71–99) are formed in the intermediate and transition states, but strands 1 and 5, located at the edges of β-sheet, are not[10-13]. **c** Folding of ribonuclease HI (RNase HI). Within 14 ms of refolding, an intermediate accumulates in which helices A, B, C, and D and strands 4 and 5 are formed[14-16]. **d** Folding of dihydrofolate reductase (DHFR). The adenosine-binding domain (ABD) attains a native-like compactness in the I_6 intermediate, while the discontinuous loop domain (DLD) becomes compact in the I_5 intermediate[19,20]. Both domains fold further to form the I_1–I_4 intermediates[18]. Finally, they fold into the native state through the four parallel channels via docking of both domains[17]. **e** Folding of α-subunit of tryptophan synthase (αTS). αTS accumulates on-pathway intermediates I_1 in two parallel pathways, in which strands 1–4 and helices 1 and 8 are formed[21-23]. Off-pathway intermediates I_{BP} are also formed early in folding. I_{BP} require unfolding before folding to native state. Pathways involving *trans*-to-*cis* Pro isomerization are ignored. **f** Folding of indole-3-glycerol phosphate synthase (IGPS). IGPS accumulates on-pathway intermediates I_1 in two parallel pathways, in which strands 2–6 and helices 2–5 are formed[24]. Off-pathway intermediates I_{BP} are also formed early in folding. I_{BP} require unfolding before folding to native state. Pathways involving *trans*-to-*cis* Pro isomerization are ignored.

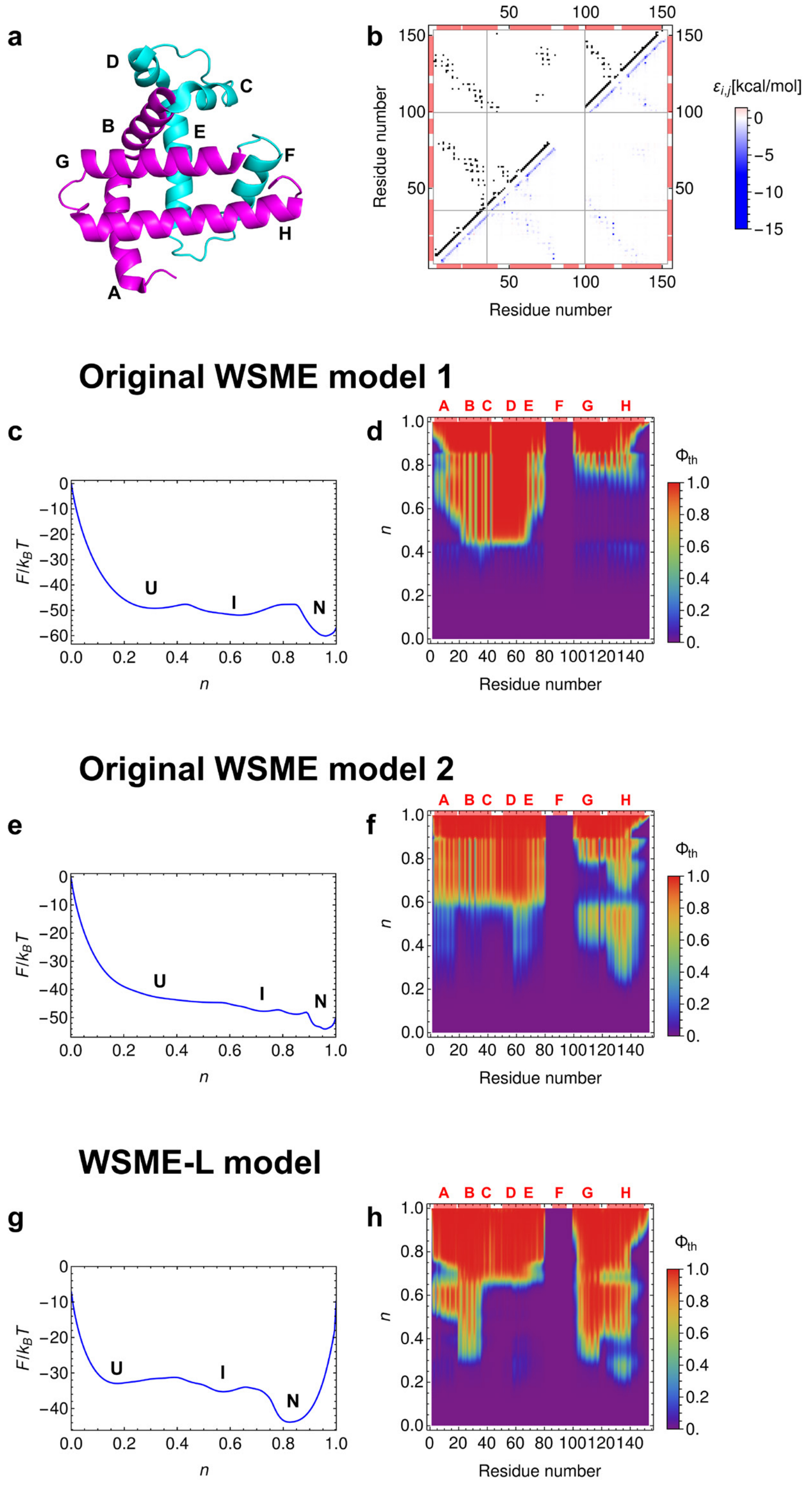

Supplementary Fig. 9. Folding of apomyoglobin (apoMb). I.
a Native structure of myoglobin. A, B, G, and H helices (residues 1–35 and 100–153) are shown in magenta, and C, D, E, and F helices (residues 36–99) are shown in cyan. **b** Contact map (top left) and AMBER-derived contact energy (bottom right). **c, e, g** One-dimensional free energy landscapes. U, I, and N denote unfolded, intermediate, and native states, respectively. **d, f, h** Residue-specific structure formation predicted by theoretical Φ-value analysis. Red and green boxes on top frame indicate locations of helices and strands, respectively, and their names are shown in the corresponding colors. Data in Panels **c–d**, **e–f**, and **g–h** were predicted using Original models 1 and 2 and WSME-L model, respectively. Source data are provided as a Source Data file.

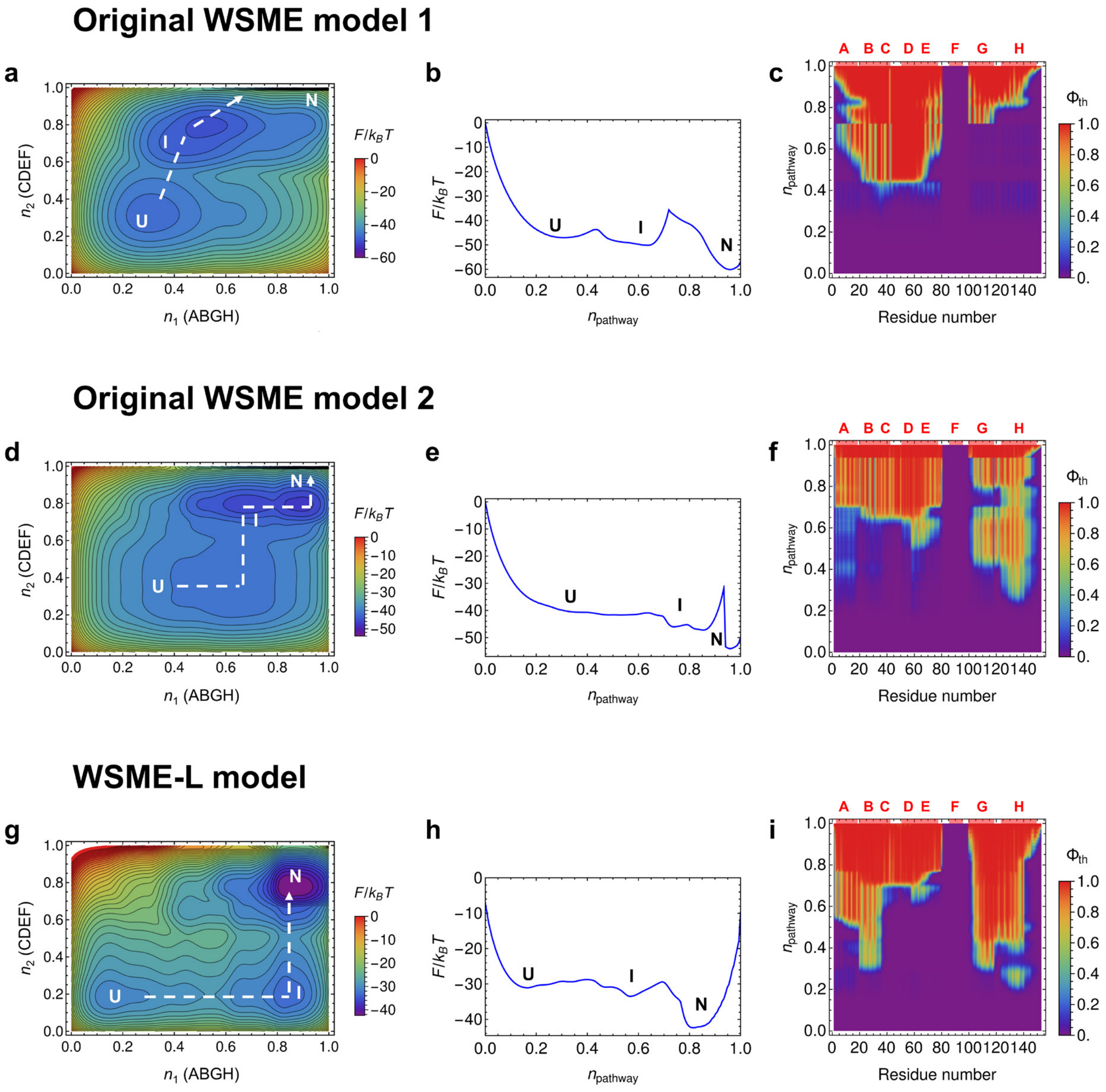

Supplementary Fig. 10. Folding of apomyoglobin (apoMb). II.
a, d, g Two-dimensional (2D) free energy landscapes. n_1 and n_2 are order parameters for magenta and cyan regions, respectively, as shown in Supplementary Fig. 9a. U, I, and N denote the unfolded, intermediate, native states, respectively. Dominant folding pathway is indicated by white dashed line. **b, e, h** Cross-sections of 2D free energy landscape along dominant folding pathway (order parameter $n_{pathway}$). **c, f, i** Residue-specific structure formation along dominant folding pathway predicted by theoretical Φ-value analysis. Red and green boxes on top frame indicate locations of helices and strands, respectively, and their names are shown in the corresponding colors. Data in Panels **a–c**, **d–f**, and **g–i** were predicted using Original models 1 and 2 and WSME-L model, respectively. Source data are provided as a Source Data file.

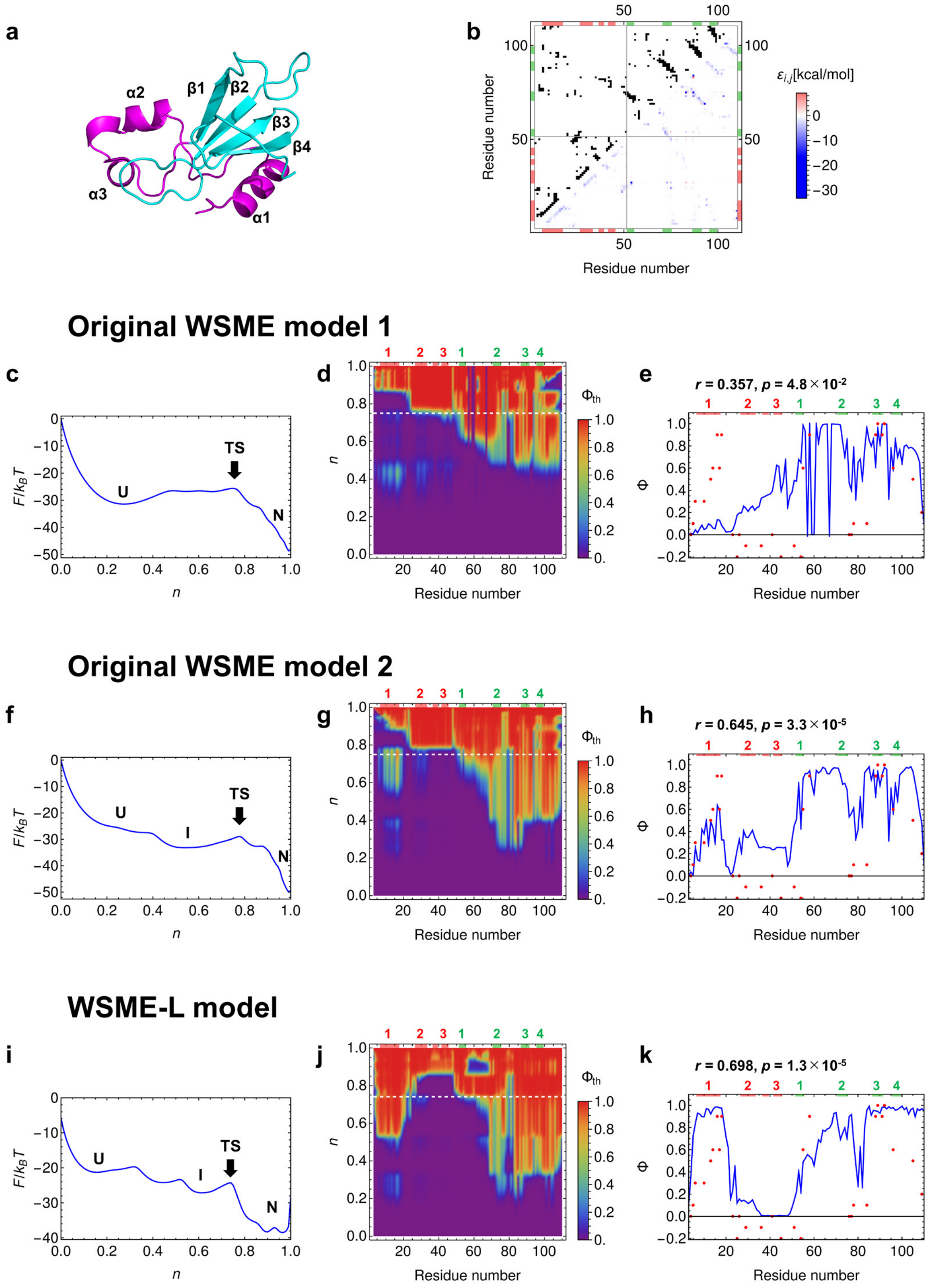

Supplementary Fig. 11. Folding of barnase. I.

a Native structure. N-terminal (residues 3–51) and C-terminal (residues 52–110) halves are shown in magenta and cyan, respectively. **b** Contact map (top left) and AMBER-derived contact energy (bottom right). **c, f, i** One-dimensional free energy landscapes. U, TS, and N denotes unfolded, transition, and native states, respectively. **d, g, j** Residue-specific structure formation predicted by theoretical Φ-value analysis. Red and green boxes on top frame indicate locations of helices and strands, respectively, and their names are shown in the corresponding colors. **e, h, k** Theoretical Φ-values in transition state (blue line) and experimental Φ-values (red filled circles). Correlation coefficients between them, *r*, and *p*-values of two-sided *t*-test without adjustments are shown. Data in Panels **c–e**, **f–h**, and **i–k** were predicted by Original models 1 and 2 and WSME-L model, respectively. Source data are provided as a Source Data file.

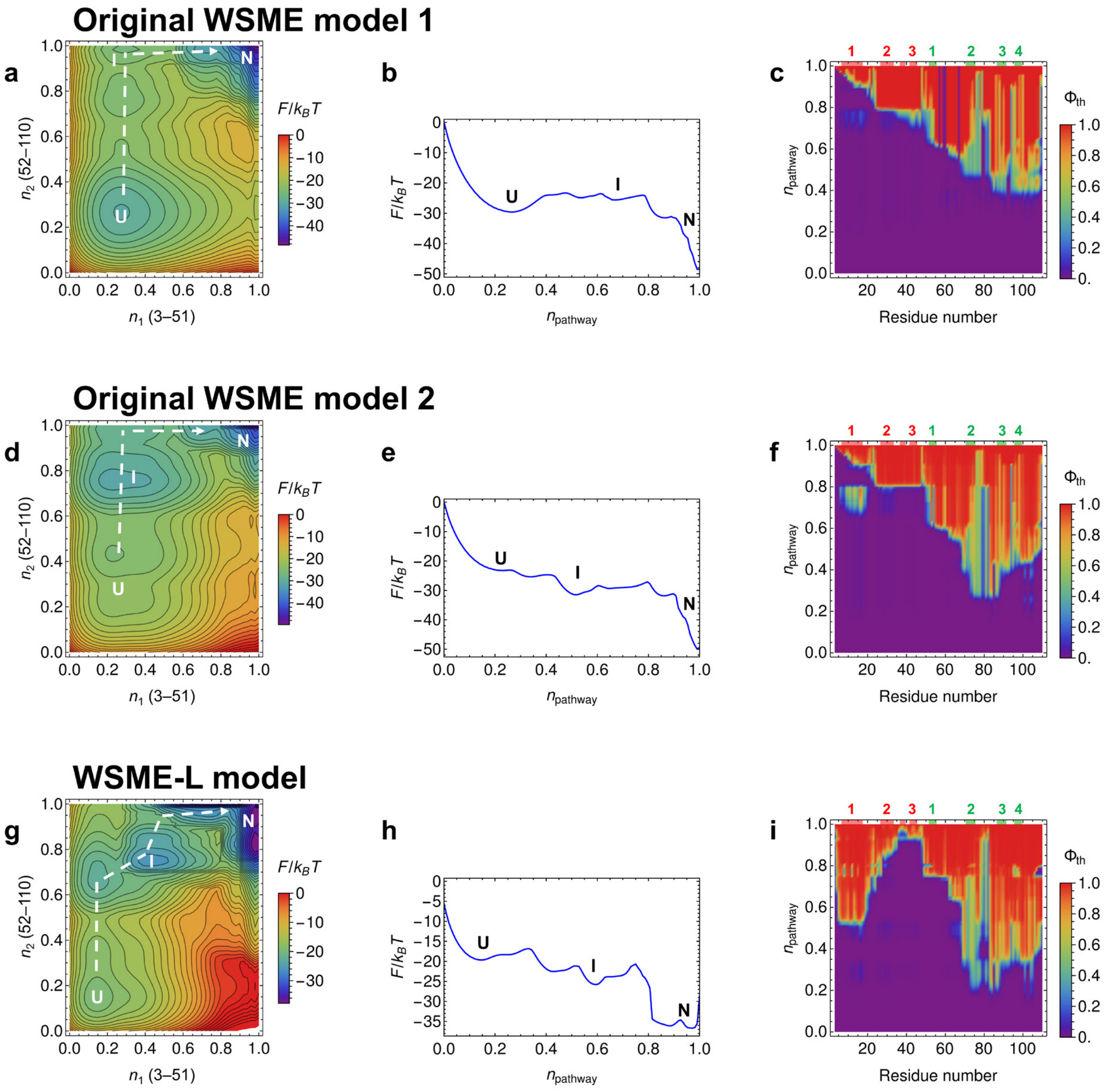

Supplementary Fig. 12. Folding of barnase. II.

a–i Details are shown in Supplementary Fig. 10. n_1 and n_2 are order parameters for magenta and cyan regions, respectively, as shown in Supplementary Fig. 11a. Source data are provided as a Source Data file.

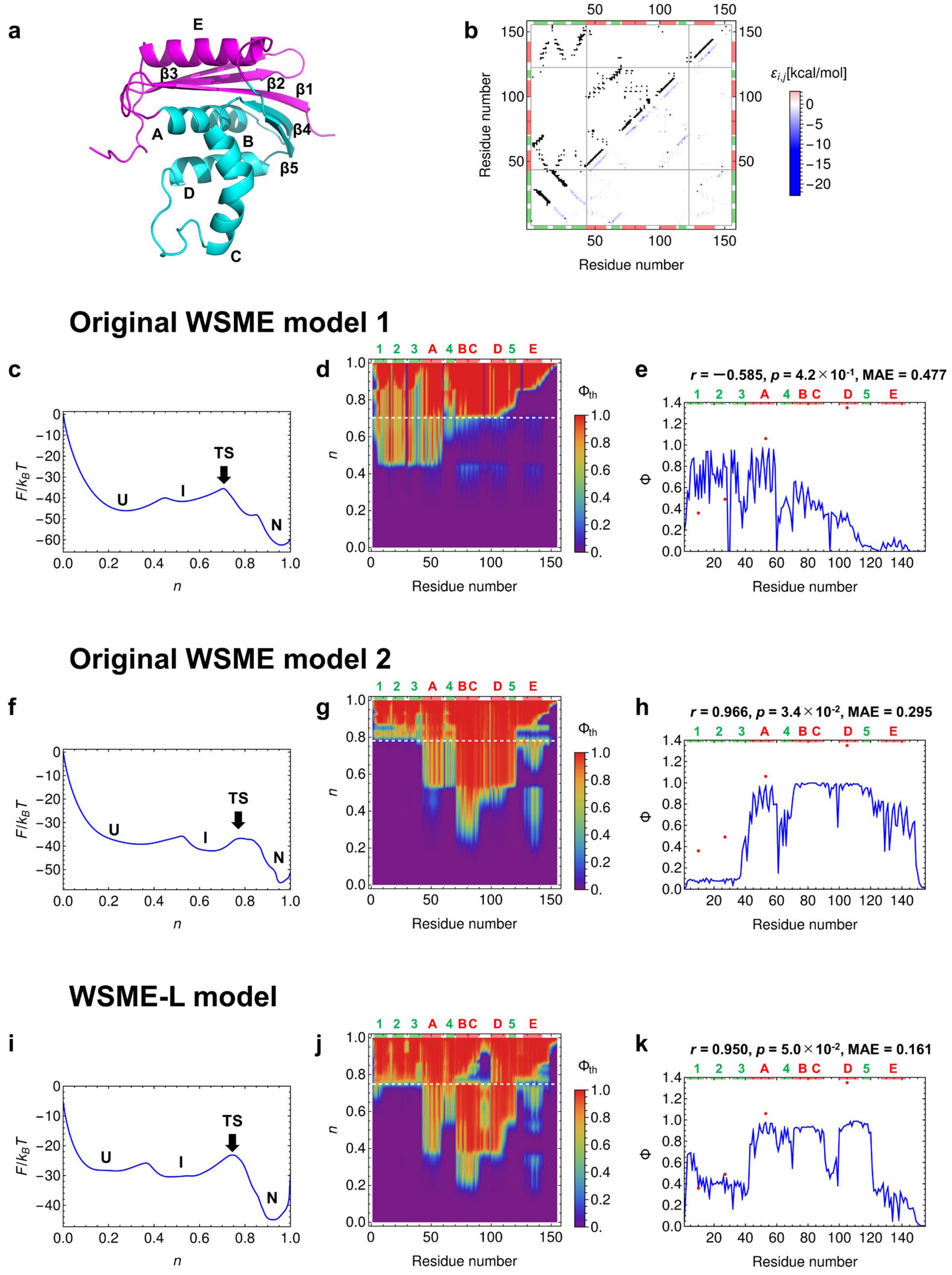

Supplementary Fig. 13. Folding of ribonuclease HI (RNase HI). I.
a–k Details are shown in Supplementary Fig. 11. In **a**, residues 1–43 and 123–155 are shown in magenta, and residues 44–122 are shown in cyan. In **e**, **h**, **k**, values predicted by WSME-L model were more consistent with the experimental ones, as indicated by the smallest mean absolute error (MAE). Source data are provided as a Source Data file.

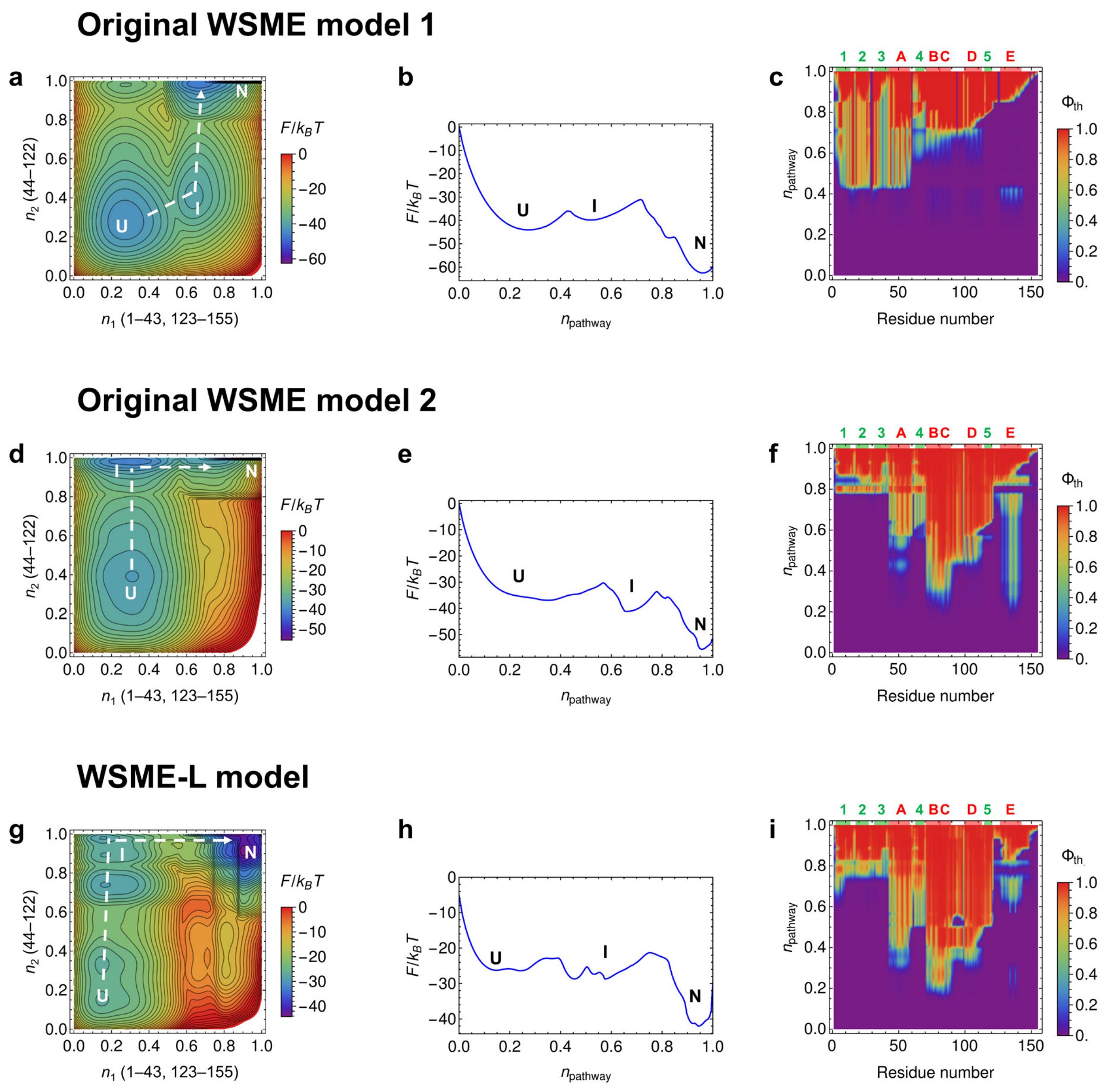

Supplementary Fig. 14. Folding of ribonuclease HI (RNase HI). II.
a–i Details are shown in Supplementary Fig. 10. n_1 and n_2 are order parameters for magenta and cyan regions, respectively, as shown in Supplementary Fig. 13a. Source data are provided as a Source Data file.

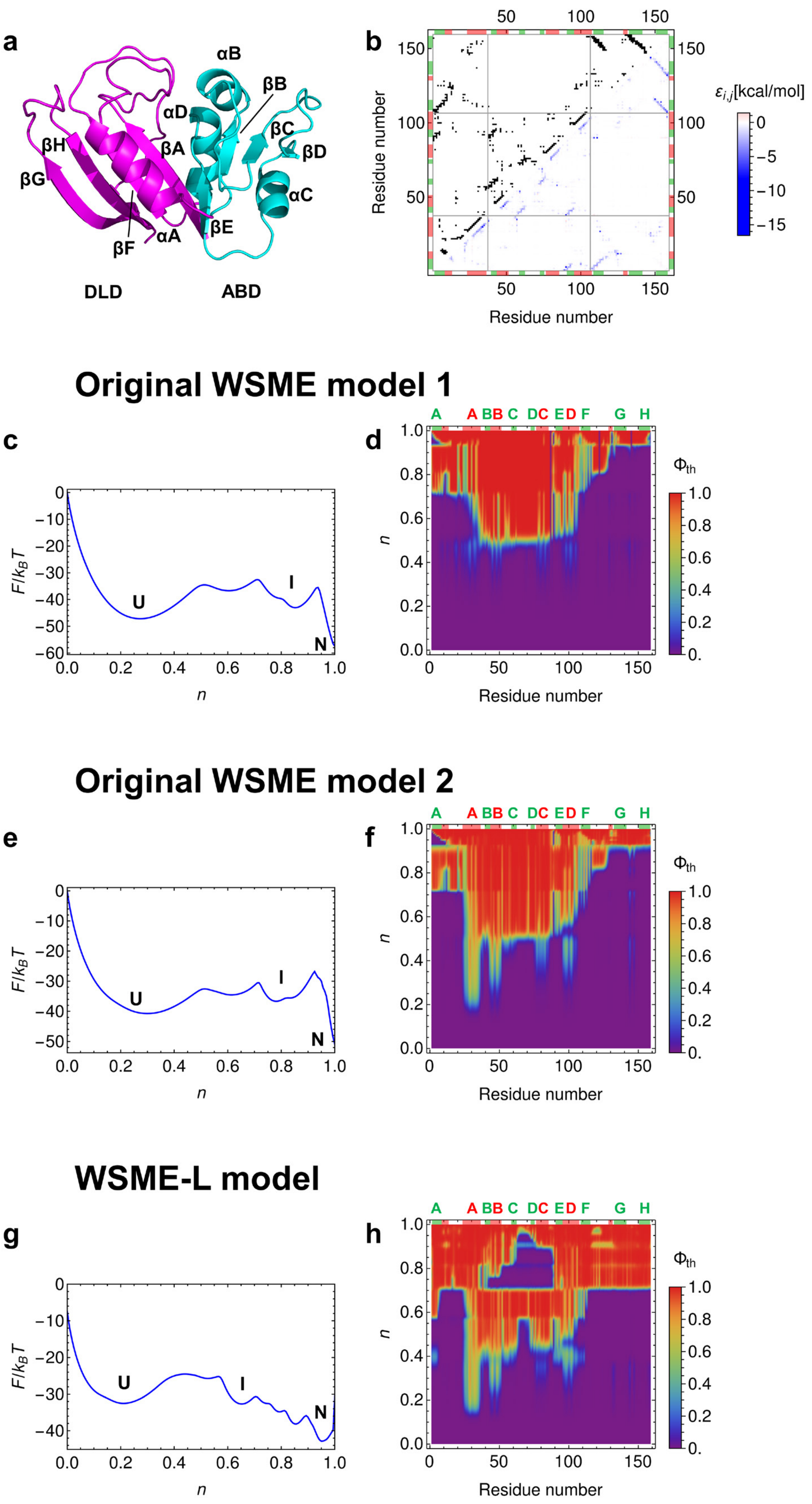

Supplementary Fig. 15. Folding of dihydrofolate reductase (DHFR). I.
a–h Details are shown in Supplementary Fig. 9. In **a** The discontinuous loop domain (DLD, residues 1–37 and 107–159) and adenosine-binding domain (ABD, residues 38–106) are shown in magenta and cyan, respectively. Source data are provided as a Source Data file.

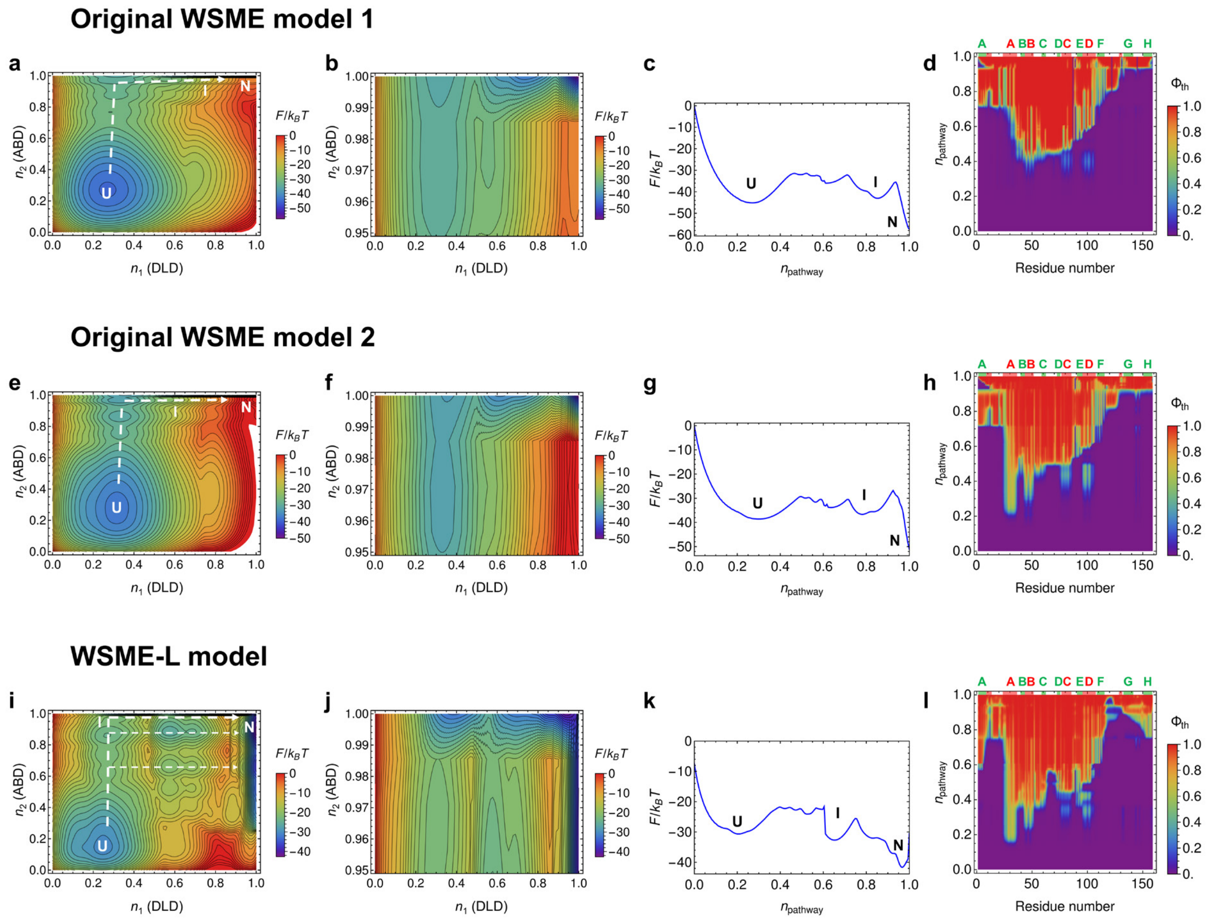

Supplementary Fig. 16. Folding of dihydrofolate reductase (DHFR). II.

a, e, i Two-dimensional (2D) free energy landscapes. n_1 and n_2 are order parameters for magenta and cyan regions, respectively, as shown in Supplementary Fig. 15a. U, I, and N denote the unfolded, intermediate, native states, respectively. Dominant folding pathway is indicated by white dashed line. In **i**, multiple folding pathways are indicated. **b, f, j** Expanded view of Panels **a**, **e**, **i**, respectively, at n_2 = 0.95–1.00, showing the presence of dominant folding pathway at n_2 = 1.00. **c**, **g**, **k** Cross-sections of 2D free energy landscape along dominant folding pathway (order parameter n_{pathway}). For WSME-L model, pathway with the highest n_2 was dominant among multiple pathways. **d, h, l** Residue-specific structure formation along dominant folding pathway predicted by theoretical Φ-value analysis. Red and green boxes on top frame indicate locations of helices and strands, respectively, and their names are shown in the corresponding colors. Data in Panels **a–d**, **e–h**, and **i–l** were predicted using Original models 1 and 2 and WSME-L model, respectively. Source data are provided as a Source Data file.

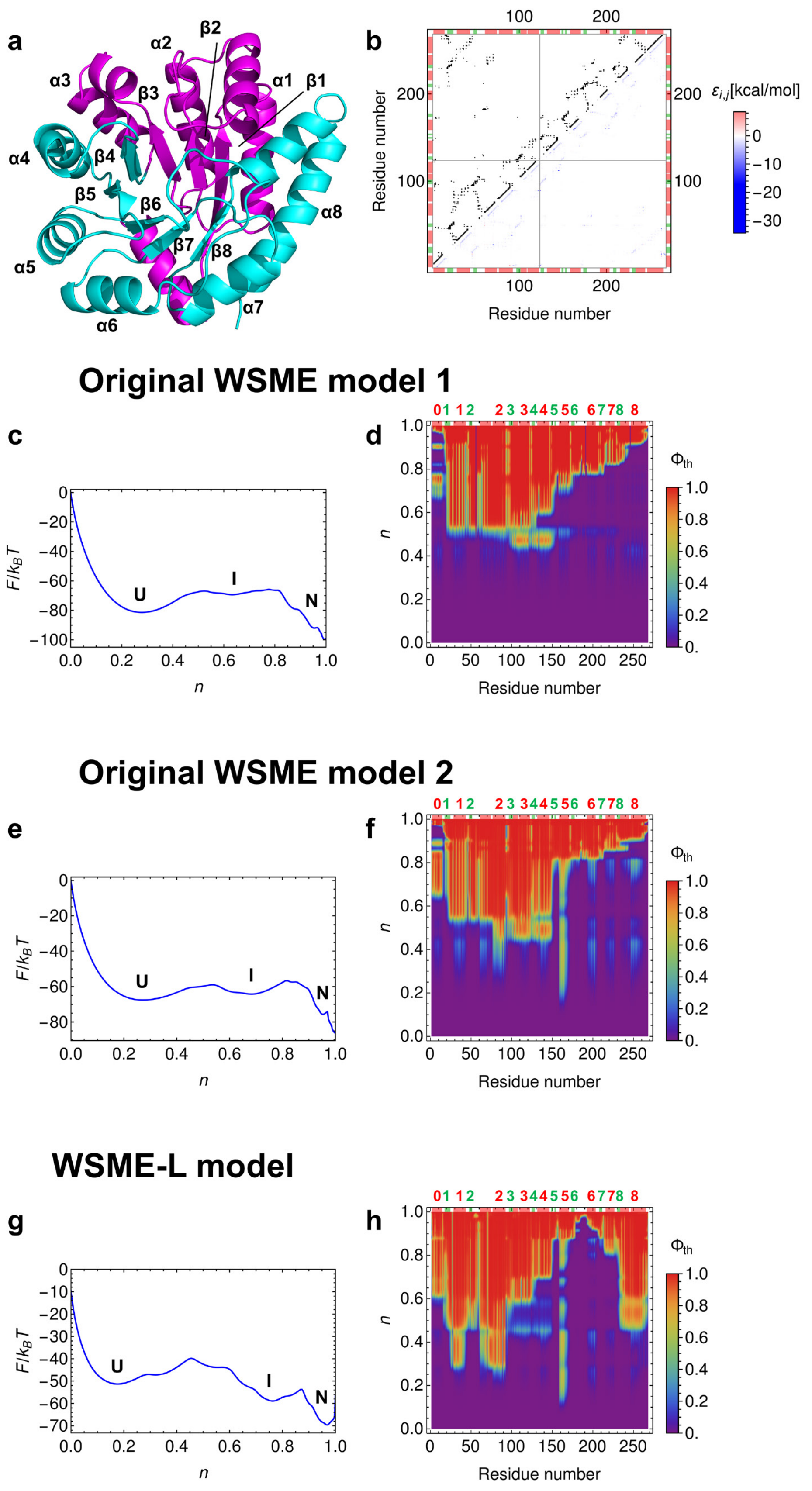

Supplementary Fig. 17. Folding of α-subunit of tryptophan synthase (αTS). I.
a–h Details are shown in Supplementary Fig. 9. In **a**, N-terminal (residues 1–123) and C-terminal (residues 124–268) halves are shown in magenta and cyan, respectively. Source data are provided as a Source Data file.

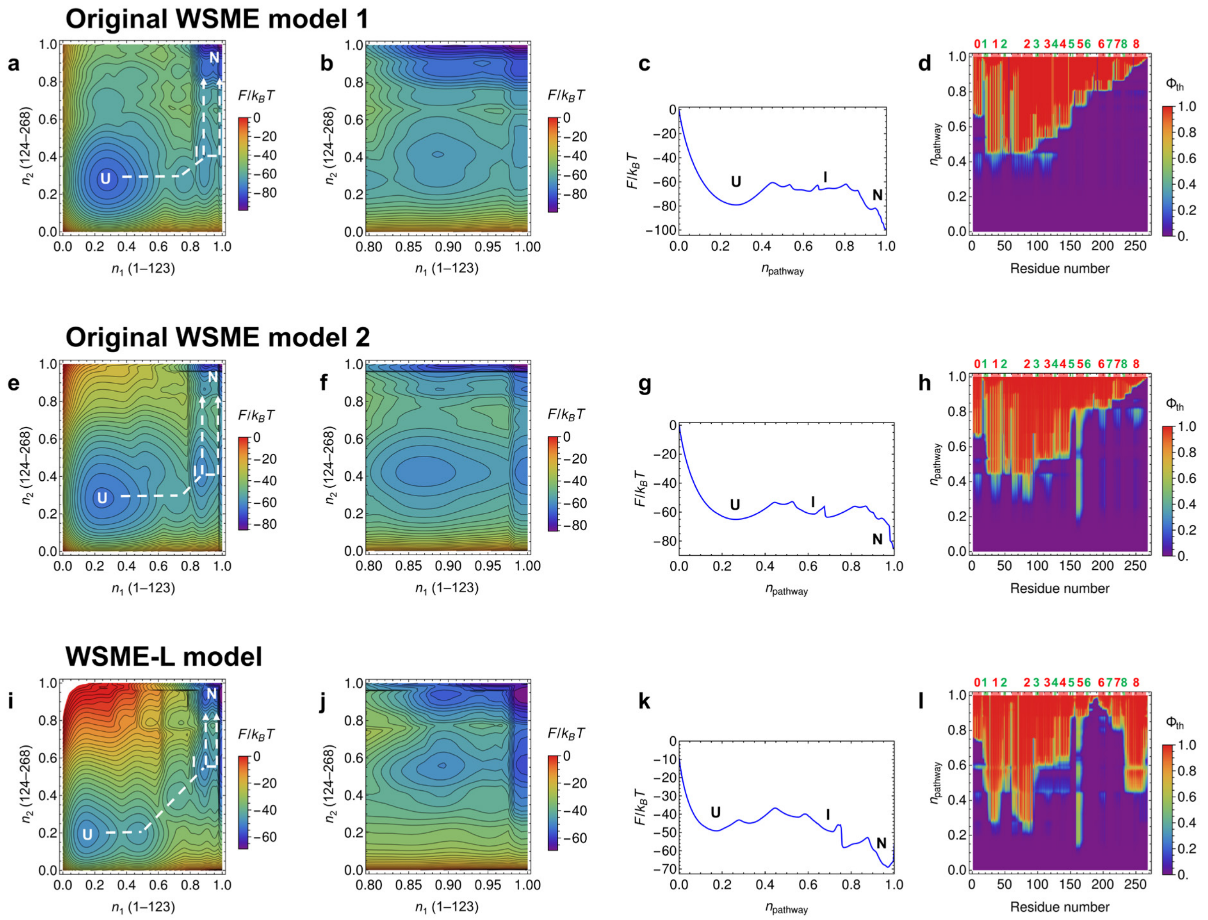

Supplementary Fig. 18. Folding of α-subunit of tryptophan synthase (αTS). II.
a–l Details are shown in Supplementary Fig. 16. n_1 and n_2 are order parameters for magenta and cyan regions, respectively, as shown in Supplementary Fig. 17a. For WSME-L model, pathway with the highest n_1 was dominant among multiple pathways. Source data are provided as a Source Data file.

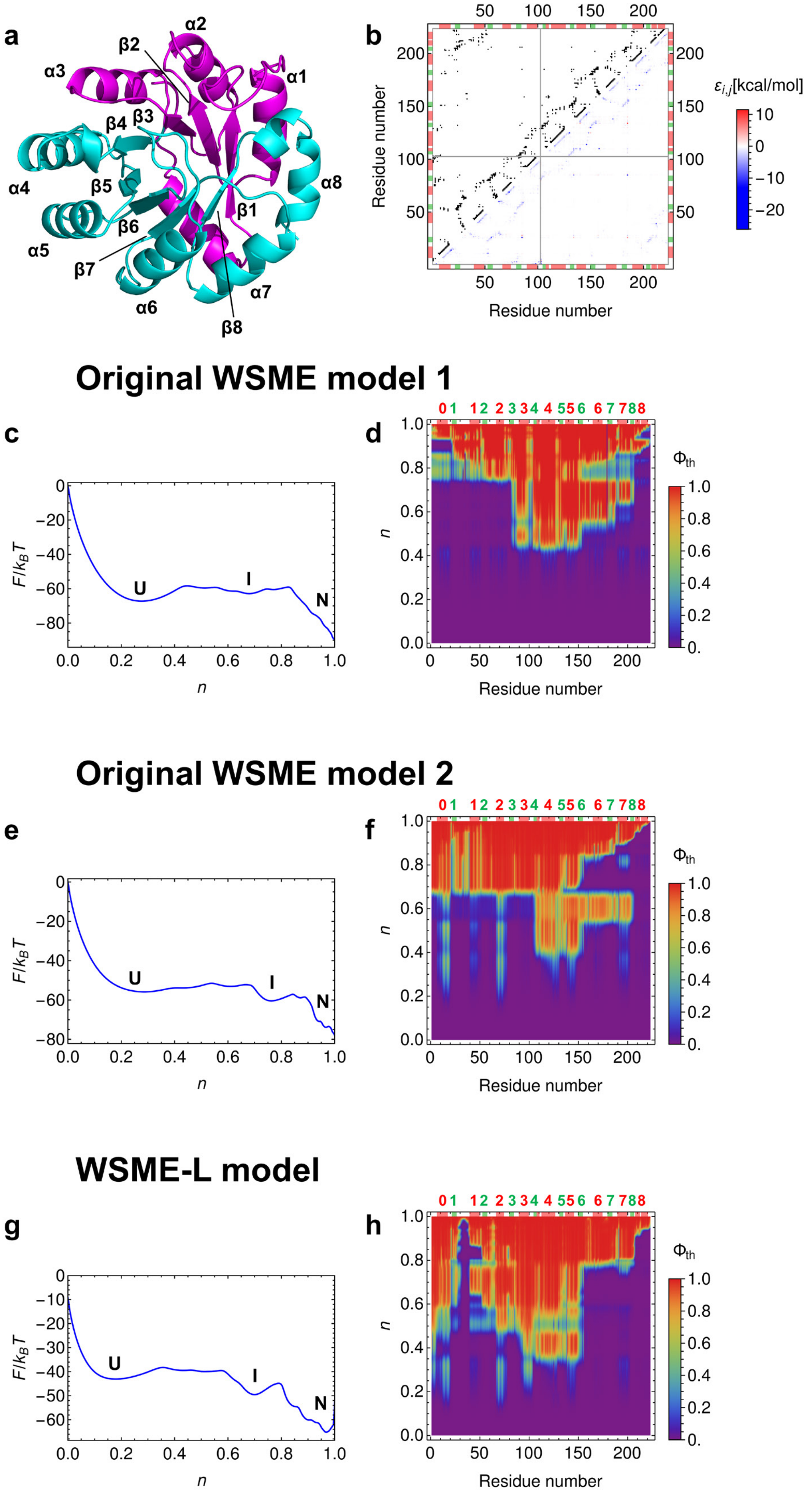

Supplementary Fig. 19. Folding of indole-3-glycerol phosphate synthase (IGPS). I.
a–h Details are shown in Supplementary Fig. 9. In **a**, N-terminal (residues 1–102) and C-terminal (residues 103–223) halves are shown in magenta and cyan, respectively. Source data are provided as a Source Data file.

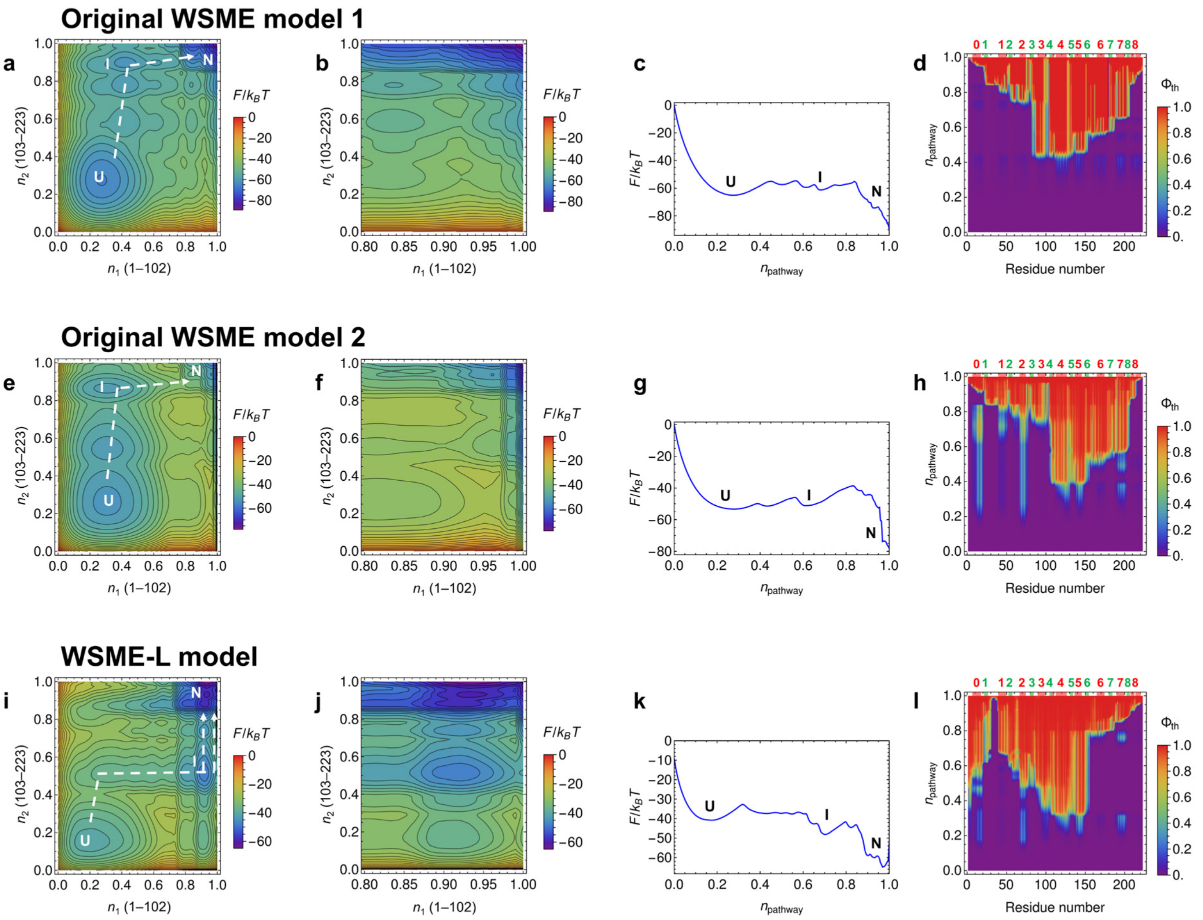

Supplementary Fig. 20. Folding of indole-3-glycerol phosphate synthase (IGPS). II.
a–l Details are shown in Supplementary Fig. 16. n_1 and n_2 are order parameters for magenta and cyan regions, respectively, as shown in Supplementary Fig. 19a. Source data are provided as a Source Data file.

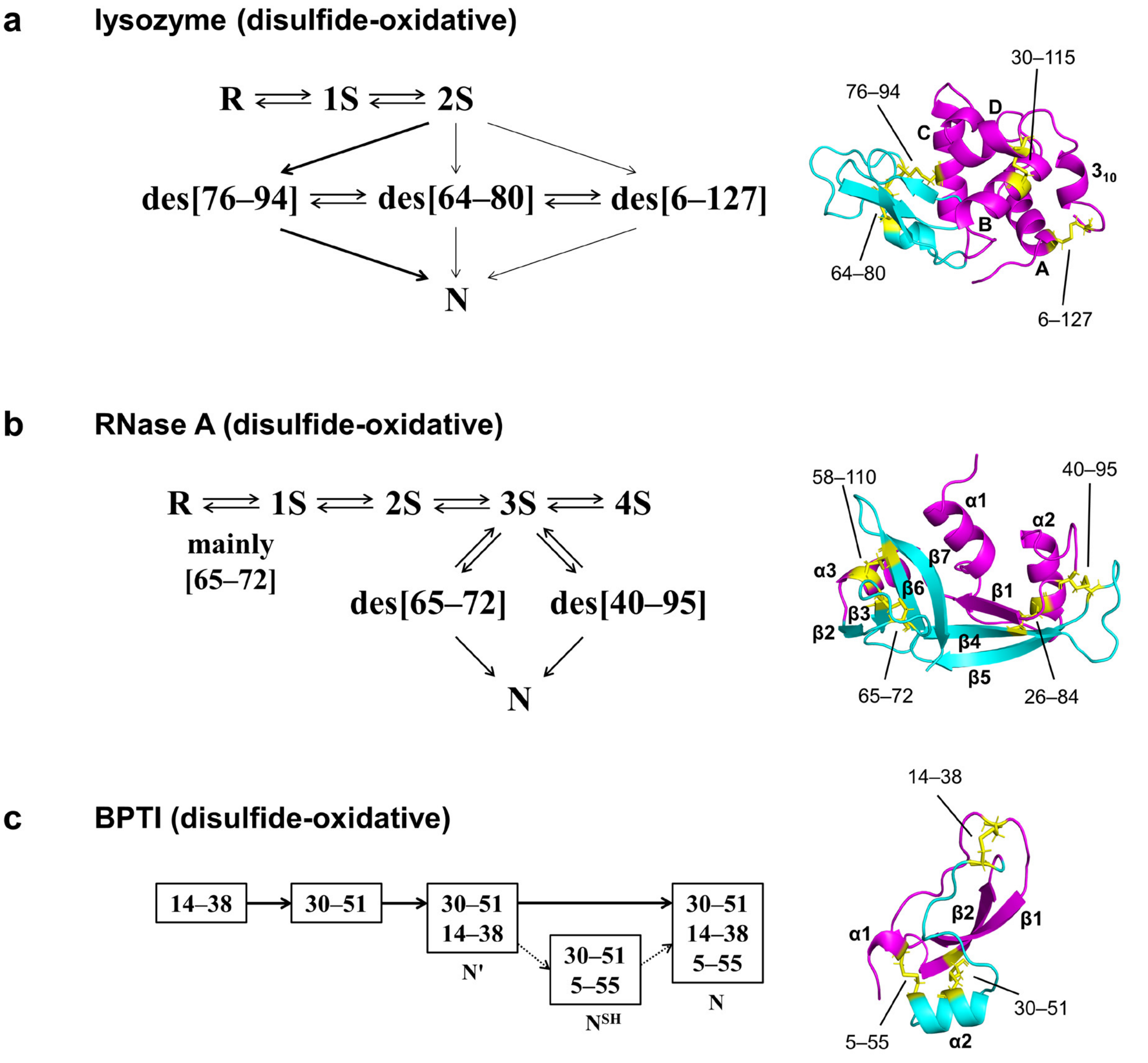

Supplementary Fig. 21. Experimentally determined protein folding pathways involving oxidative disulfide bond formation.

a Oxidative folding of hen egg-white lysozyme. R and N denote reduced and native states, respectively. 1S and 2S denote one- and two-disulfide species, respectively. Three types of three-disulfide intermediates des[76–94], des[64–80], and des[6–127] are accumulated, in which three disulfide bonds other than 76–94, 64–80, and 6–127 are formed, respectively[35,39]. Among them, des[76–94] is populated in the largest amount[39]. **b** Oxidative folding of ribonuclease A (RNase A). 3S and 4S denote three- and four-disulfide species, respectively. Formation of a 65–72 disulfide bond is strongly favored early in the folding[44]. In one pathway, however, it is disrupted in middle stage, resulting in accumulation of des[65–72], in which three disulfide bonds other than 65–72 are formed[46-49]. In the other pathway, des[40–95] accumulates, and the 40–95 disulfide bond is formed in final stage of folding[46-49]. **c** Oxidative folding of bovine pancreatic trypsin inhibitor (BPTI). In dominant pathway, a single disulfide [14–38] intermediate with a disulfide bond between residues 14 and 38 is initially formed, which then rearranges to more stable [30–51] intermediate[51,52]. A two-disulfide [30–51; 14–38] intermediate (denoted N') is then formed under conditions where glutathione concentrations are close to those in endoplasmic reticulum, followed by native structure[53]. At low concentrations of oxidized glutathione, the 14–38 disulfide bond is transiently reduced, and a two-disulfide [30–51; 5–55] intermediate (denoted N^{SH}) accumulates before forming native state[50].

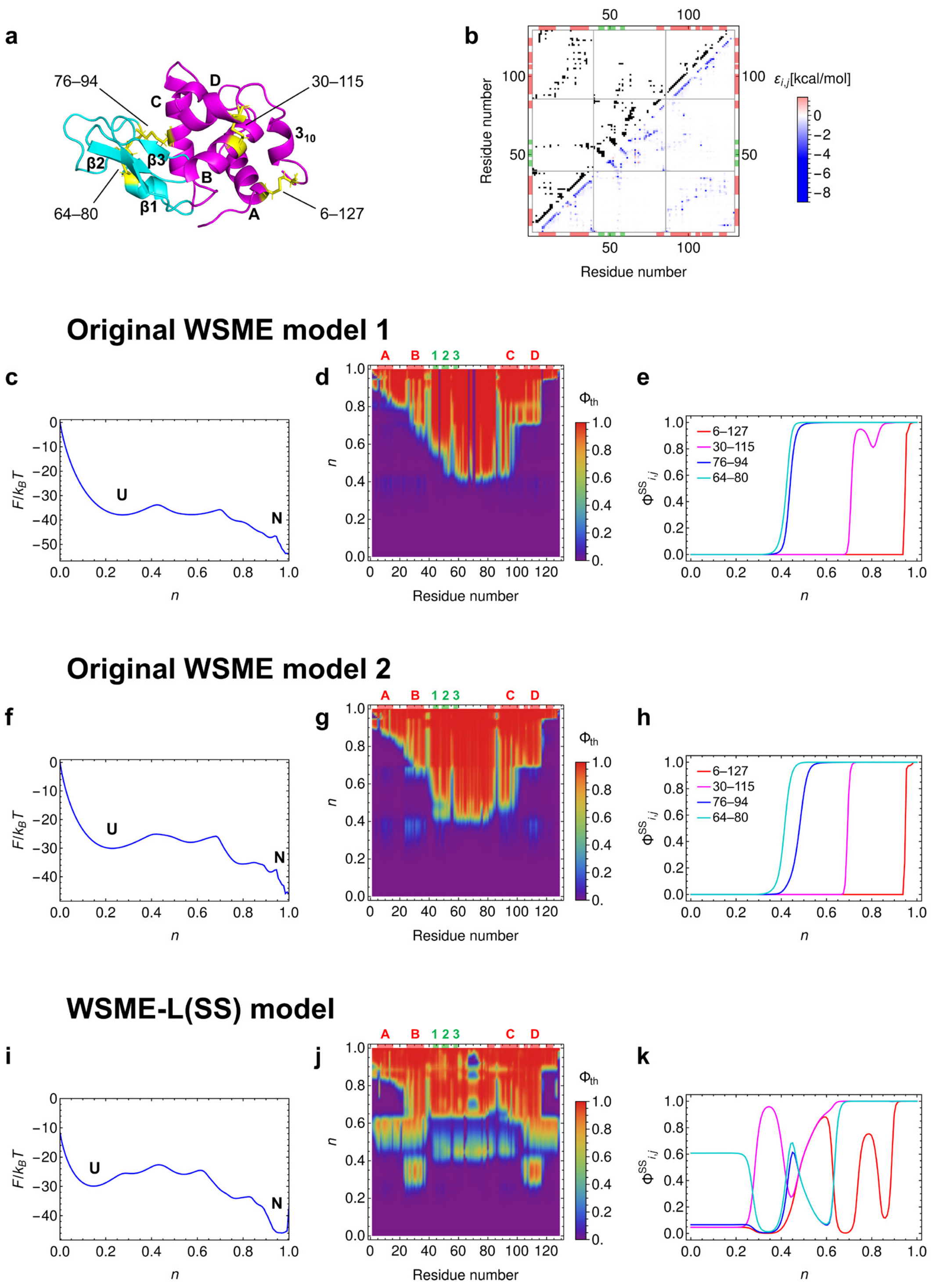

Supplementary Fig. 22. Oxidative folding of hen egg-white lysozyme. I.
a Native structure. The α-domain (residues 1–39 and 86–129) and β-domain (residues 40–85) are shown in magenta and cyan, respectively. Disulfide bonds are shown in yellow with Cys residue numbers. **b** Contact map (top left) and AMBER-derived contact energy (bottom right). **c, f, i** One-dimensional free energy landscapes. U and N denote unfolded and native states, respectively. **d, g, j** Residue-specific structure formation predicted by theoretical Φ-value analysis. Red and green boxes on top frame indicate locations of helices and strands, respectively, and their names are shown in the corresponding colors. **e, h, k** Degree of disulfide bond formation $\Phi_{i,j}^{\mathrm{SS}}$. Data in Panels **c–e**, **f–h**, and **i–k** were predicted using Original models 1 and 2 and WSME-L(SS) model, respectively. Source data are provided as a Source Data file.

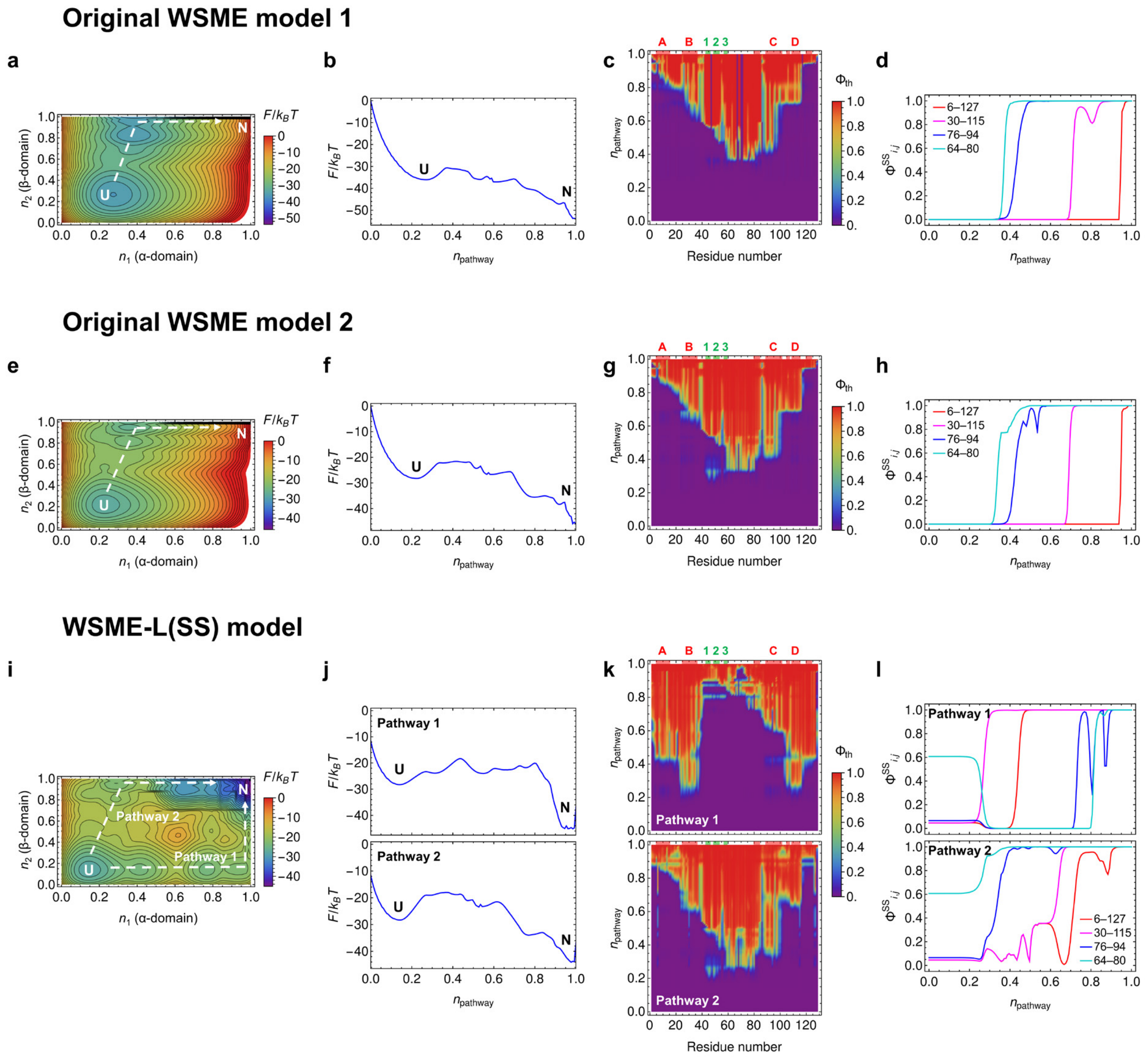

Supplementary Fig. 23. Oxidative folding of hen egg-white lysozyme. II.

a, e, i Two-dimensional (2D) free energy landscapes. n_1 and n_2 are order parameters for magenta and cyan regions, respectively, as shown in Supplementary Fig. 22a. Dominant folding pathway(s) are indicated by white dashed line(s). **b, f, j** Cross-sections of 2D free energy landscape along dominant folding pathway(s) (n_{pathway}). **c, g, k** Residue-specific structure formation along dominant folding pathway(s) predicted by theoretical Φ-value analysis. Red and green boxes on top frame indicate locations of helices and strands, respectively, and their names are shown in the corresponding colors. **d, h, l** Degree of disulfide bond formation $\Phi^{SS}_{i,j}$ along dominant folding pathway(s). Data in Panels **a–d**, **e–h**, and **i–l** were predicted using Original models 1 and 2 and WSME-L(SS) model, respectively. Source data are provided as a Source Data file.

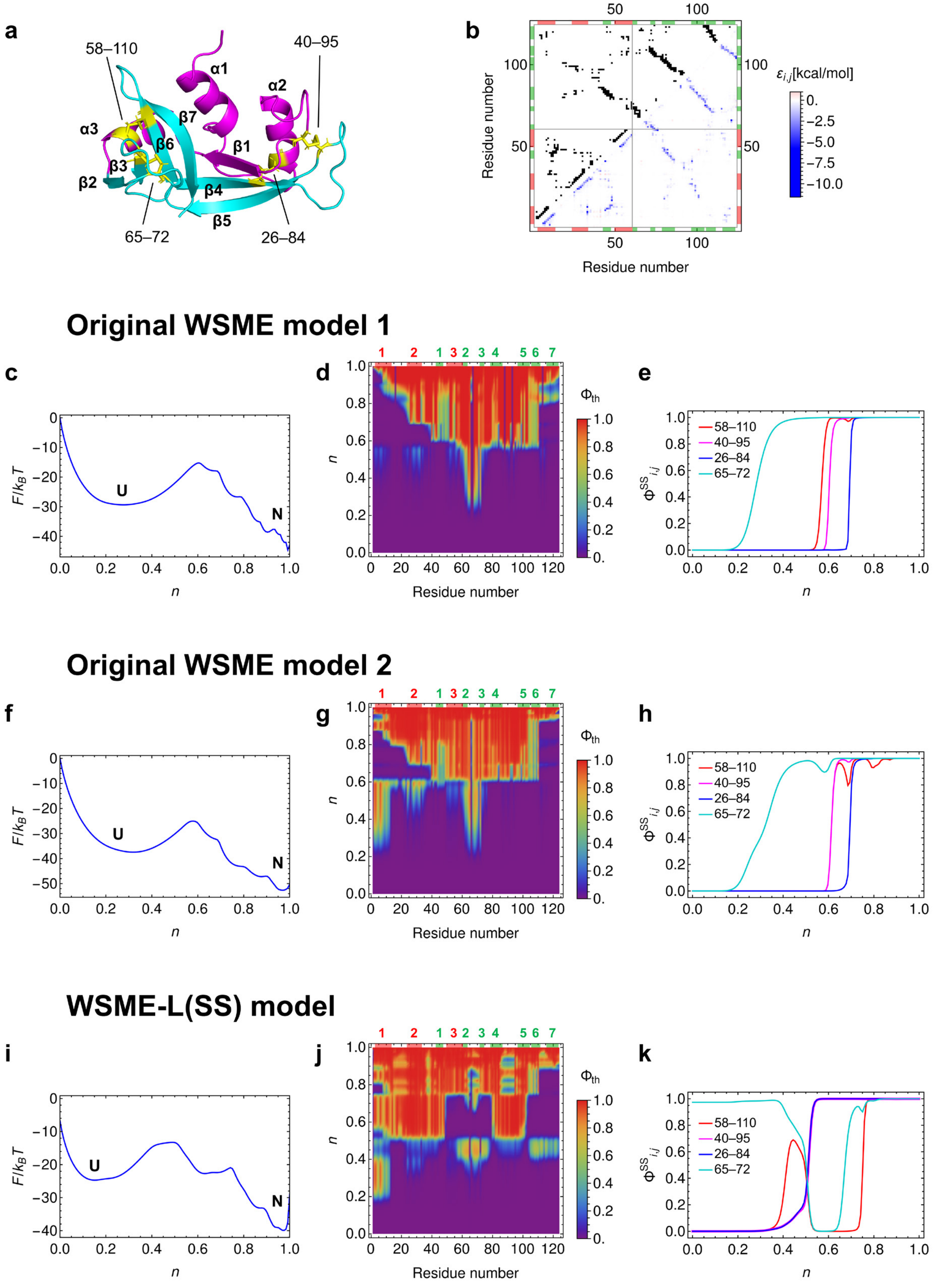

Supplementary Fig. 24. Oxidative folding of ribonuclease A (RNase A). I.
a–k Details are shown in Supplementary Fig. 22. In **a**, N-terminal (residues 1–60) and C-terminal (residues 61–124) halves are shown in magenta and cyan, respectively. Source data are provided as a Source Data file.

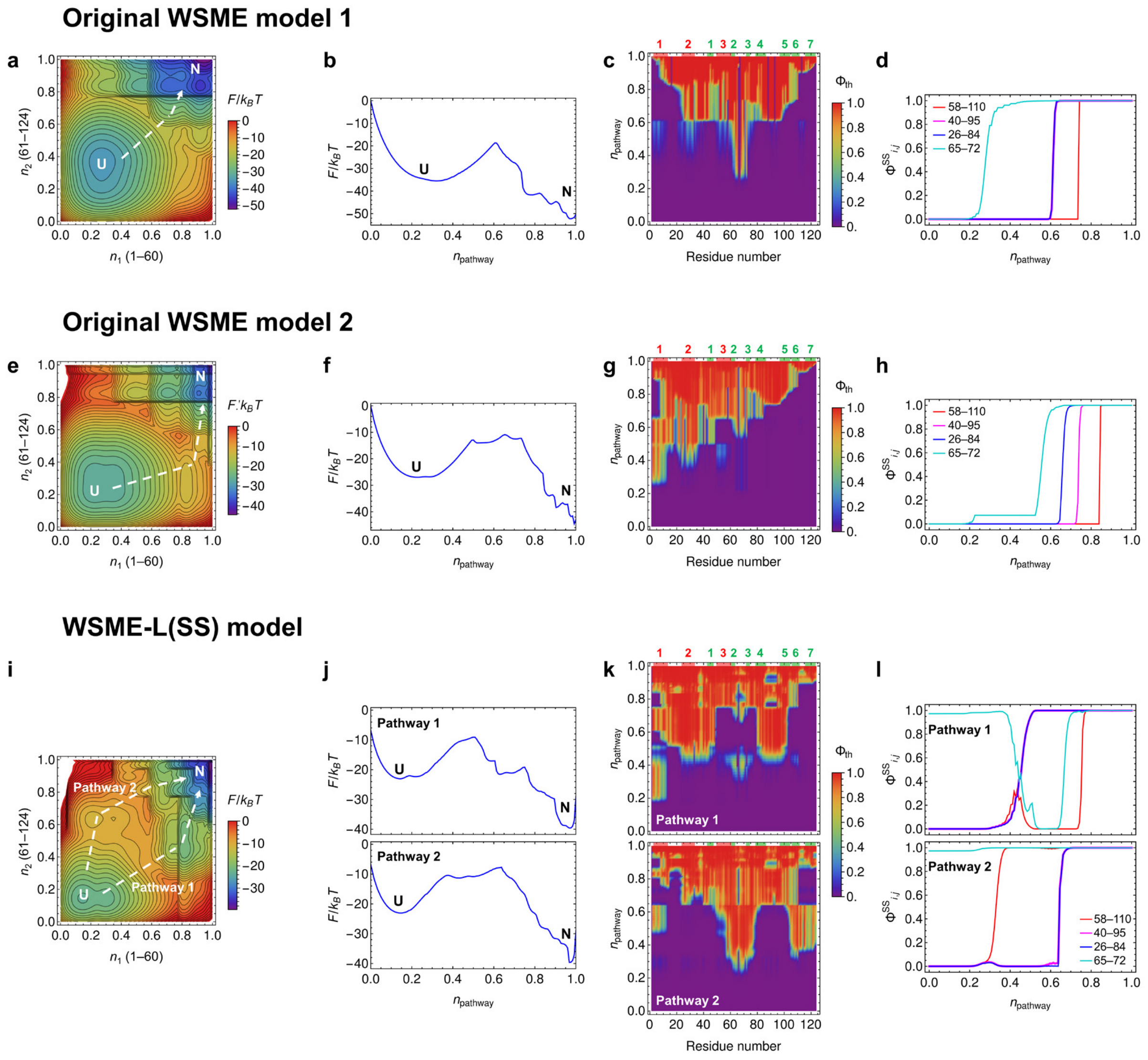

Supplementary Fig. 25. Oxidative folding of ribonuclease A (RNase A). II.
a–l Details are shown in Supplementary Fig. 23. n_1 and n_2 are order parameters for magenta and cyan regions, respectively, as shown in Supplementary Fig. 24a. Source data are provided as a Source Data file.

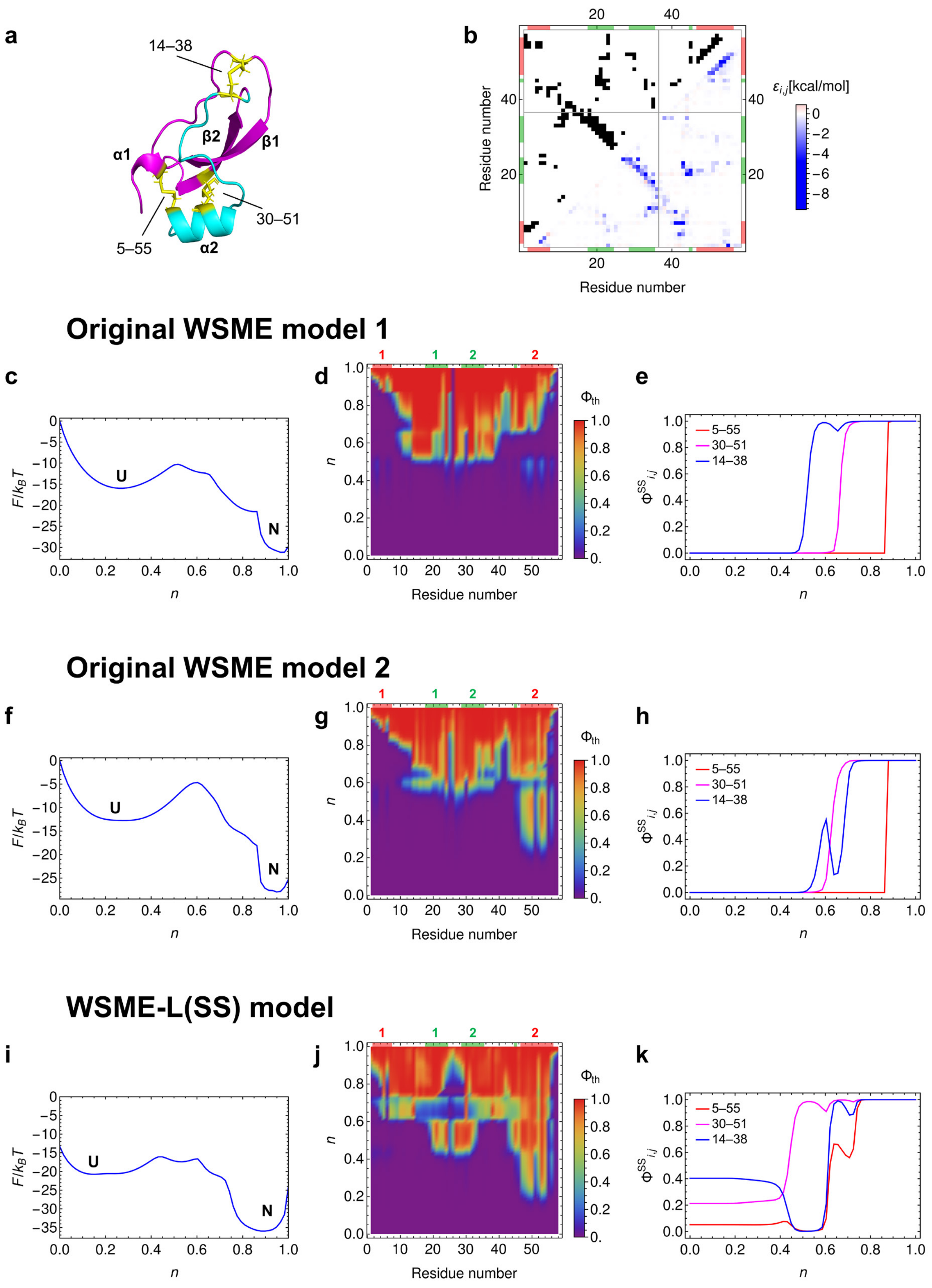

Supplementary Fig. 26. Oxidative folding of bovine pancreatic trypsin inhibitor (BPTI). I.

a–k Details are shown in Supplementary Fig. 22. In **a**, N-terminal (residues 1–36) and C-terminal (residues 37–58) regions are shown in magenta and cyan, respectively. Source data are provided as a Source Data file.

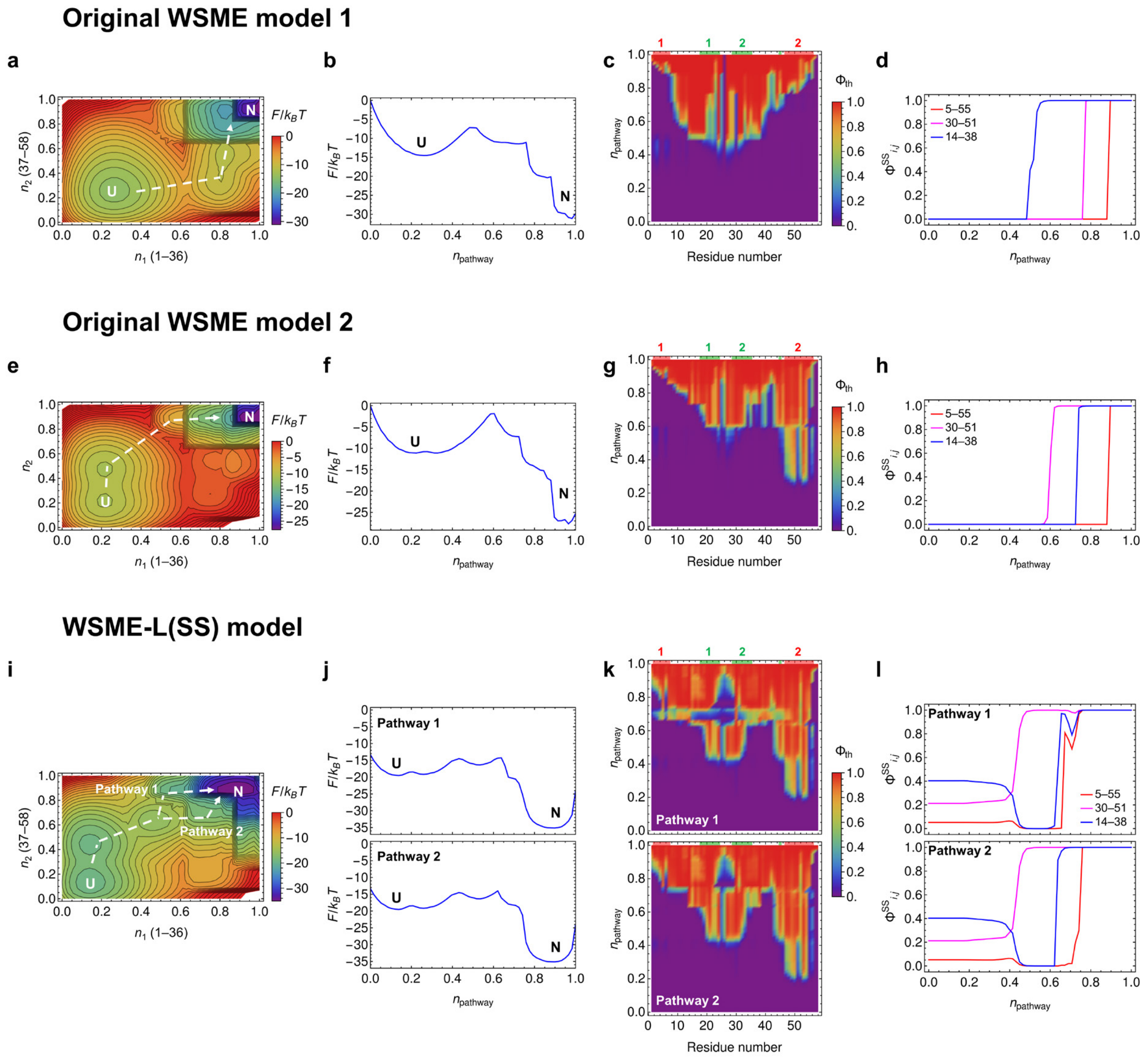

Supplementary Fig. 27. Oxidative folding of bovine pancreatic trypsin inhibitor (BPTI). II.

a–l Details are shown in Supplementary Fig. 23. n_1 and n_2 are order parameters for magenta and cyan regions, respectively, as shown in Supplementary Fig. 26a. Source data are provided as a Source Data file.

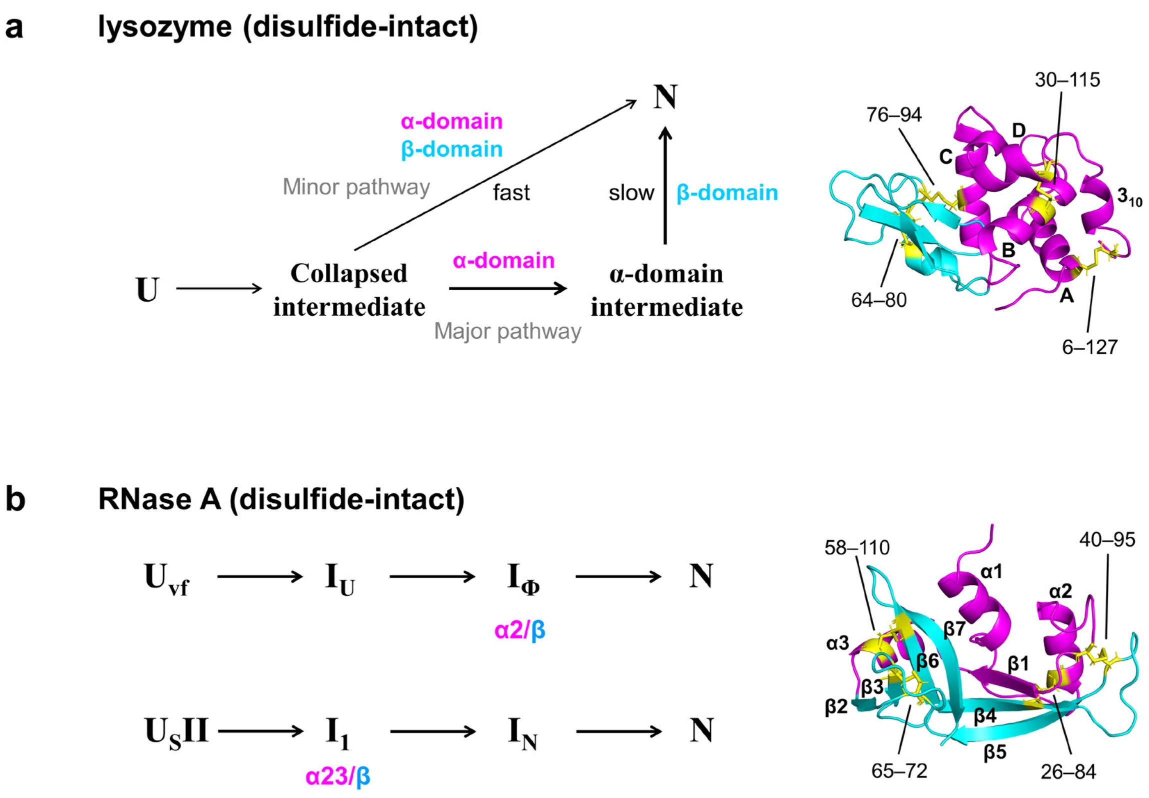

Supplementary Fig. 28. Experimentally determined folding pathways of disulfide-intact proteins.

a Folding of disulfide-intact lysozyme[25-34,36,37]. Lysozyme exhibits complex folding behavior with collapsed intermediate and two subsequent parallel pathways. Collapsed intermediate is formed within milliseconds. The major slow folding pathway accumulates α-domain intermediate in which α-domain is formed but not β-domain. In the minor fast folding pathway, collapsed intermediate folds directly to native state with simultaneous formation of α- and β-domains. **b** Folding of disulfide-intact ribonuclease A (RNase A). The major slow pathway is the folding from U_SII unfolded state where Pro93 and Pro114 that adopt *cis* conformations in native state are in *trans* conformations. In I_1 intermediate, helices 2 and 3 (residues 24–34 and 50–60, respectively) and β-sheet are stabilized[40,41]. In contrast, RNase A folding is very fast when starting from U_{vf} unfolded state where both Pro93 and Pro114 are in *cis* conformations. I_U intermediate is largely unfolded[43]. In subsequent I_Φ intermediate, which is formed by hydrophobic collapse, helix 2 and a large part of β-sheet, especially residues 82–84, are formed, while helix 1 (residues 3–13), residues 50–72 including helix 3, and C-terminal region of β-sheet are unstructured[42]. These experiments were performed at pH 3–4 where Asp and Glu are protonated. Therefore, these residues were protonated when calculating AMBER-derived contact energies in this study. Since WSME-L(SS_{intact}) model does not consider non-native interactions, its prediction for Rnase A should be consistent with the folding from U_{vf}.

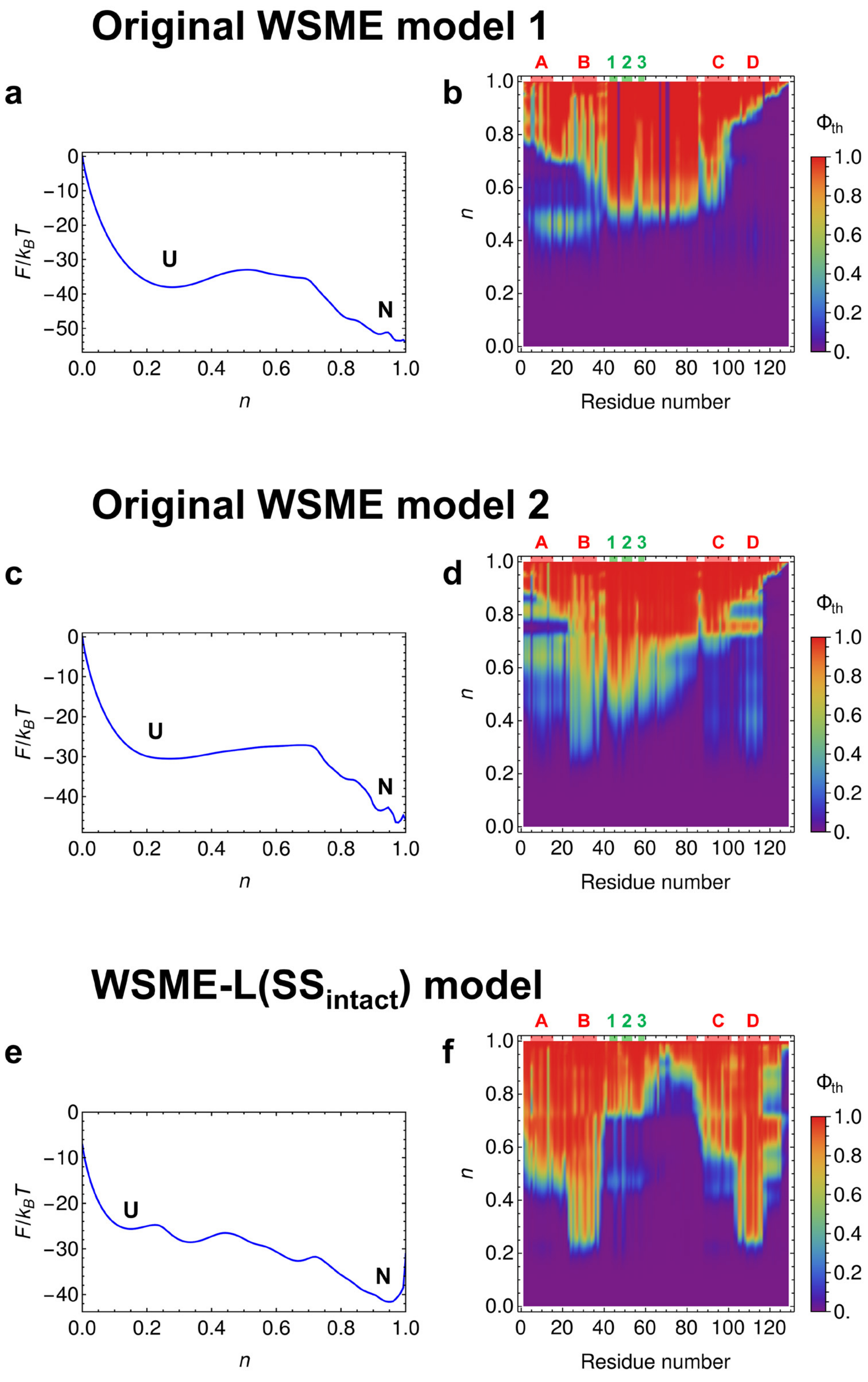

Supplementary Fig. 29. Folding of disulfide-intact hen egg-white lysozyme. I.
A, c, e One-dimensional free energy landscapes. U and N denote unfolded and native states, respectively. **b, d, f** Residue-specific structure formation predicted by theoretical Φ-value analysis. Red and green boxes on top frame indicate locations of helices and strands, respectively, and their names are shown in the corresponding colors. Data in Panels **a–b**, **c–d**, and **e–f** were predicted by Original models 1 and 2 and WSME-L(SS_{intact}) model, respectively. Source data are provided as a Source Data file.

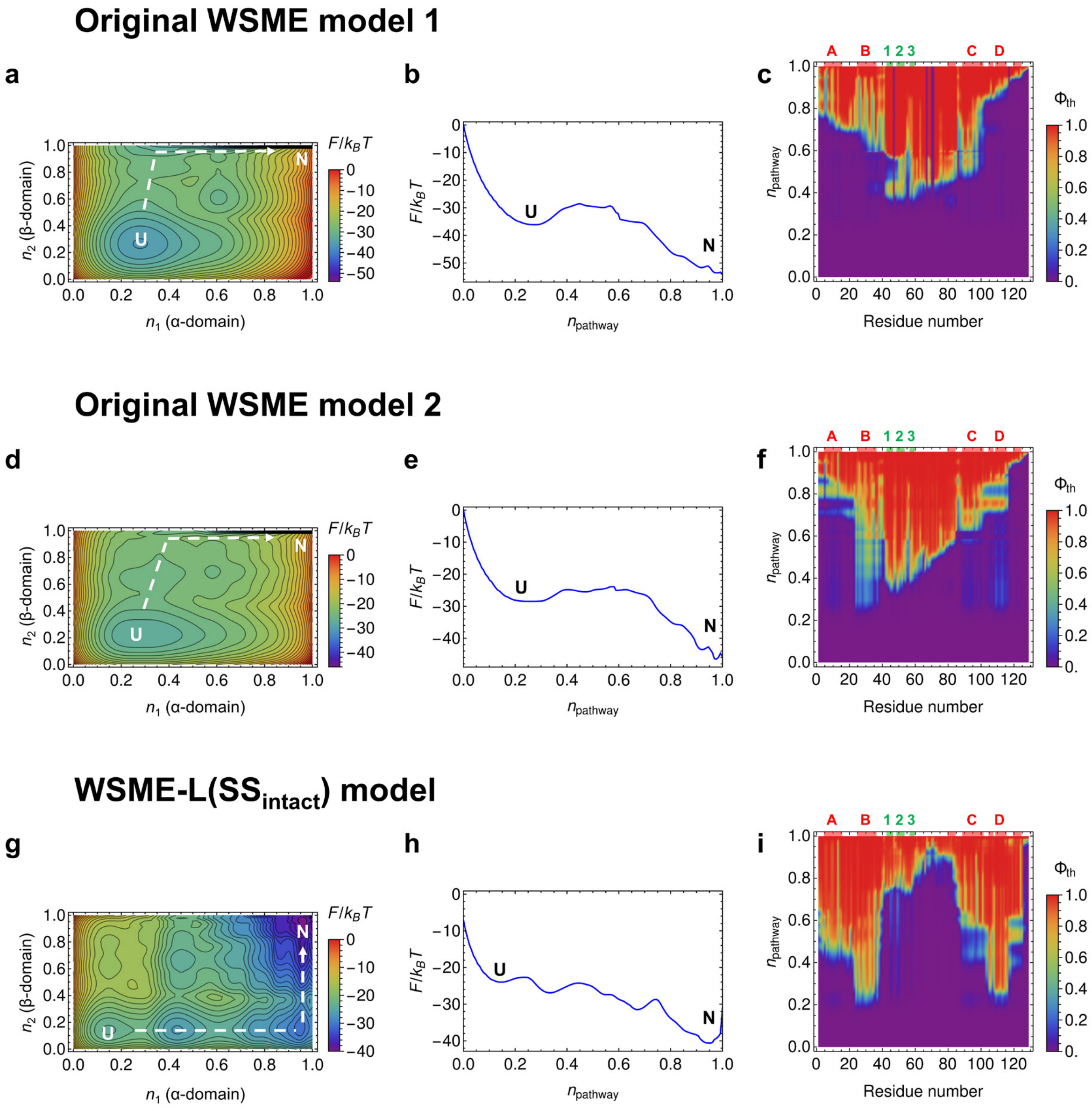

Supplementary Fig. 30. Folding of disulfide-intact hen egg-white lysozyme. II.
a–i Details are shown in Supplementary Fig. 10. n_1 and n_2 are order parameters for magenta and cyan regions, respectively, as shown in Supplementary Fig. 22a. Source data are provided as a Source Data file.

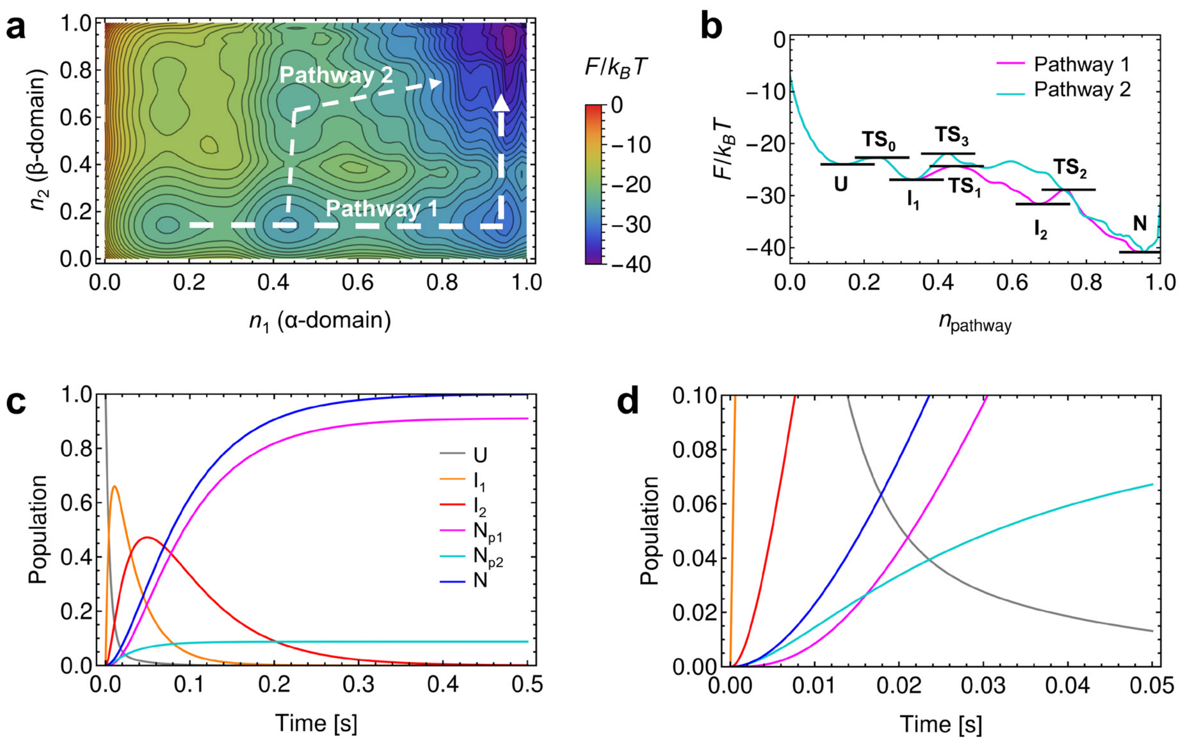

Supplementary Fig. 31. Kinetic analysis of lysozyme folding at 293 K.
a Two-dimensional (2D) free energy landscape of lysozyme predicted by WSME-L(SS_{intact}) model. Two pathways are indicated by white dashed lines. **b** Cross-sections of 2D landscape along Pathways 1 (magenta) and 2 (cyan). Free energy levels of kinetic species (U, I_1, I_2, and N) and transition states (TS_0, TS_1, TS_2, and TS_3) are highlighted by short horizontal black lines. **c** Time evolution of concentrations of kinetic species calculated from free energy values shown in **b**. Total concentration was normalized to 1. **d** Expanded view of time evolution during first 0.05 s. Color scheme is same as that shown in **c**. Source data are provided as a Source Data file.

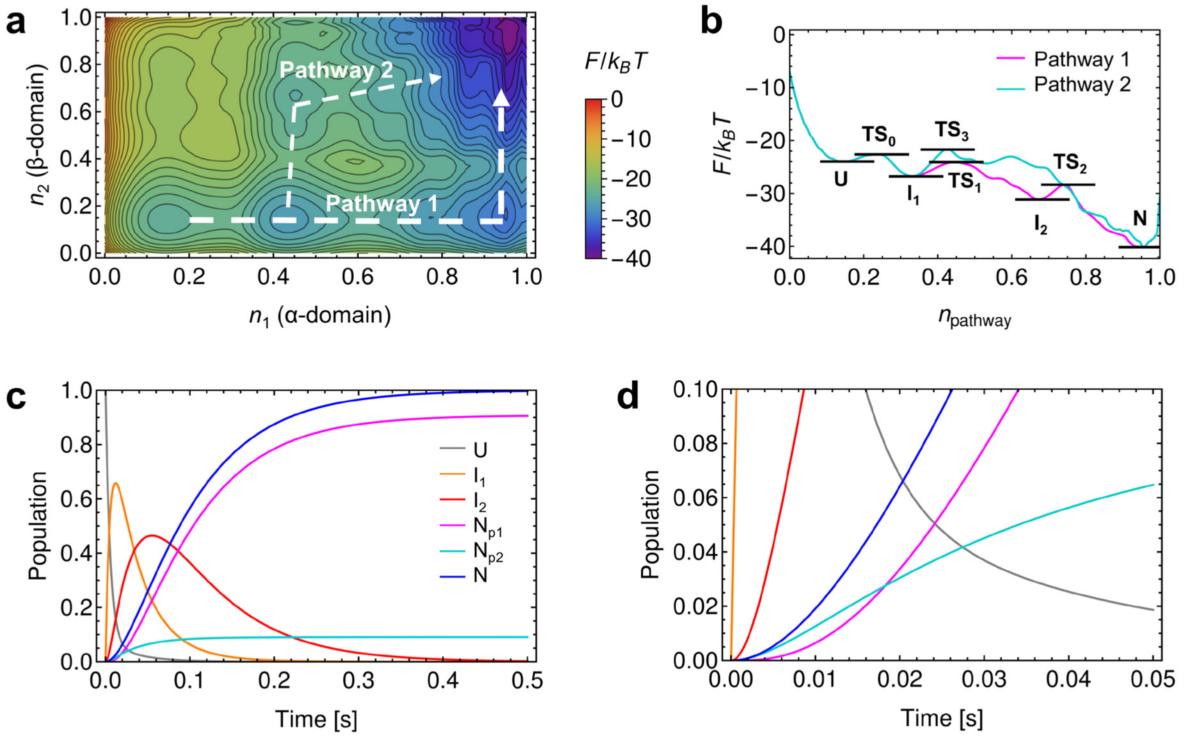

Supplementary Fig. 32. Kinetic analysis of lysozyme folding at 308 K.
a–d Details are shown in Supplementary Fig. 31. Source data are provided as a Source Data file.

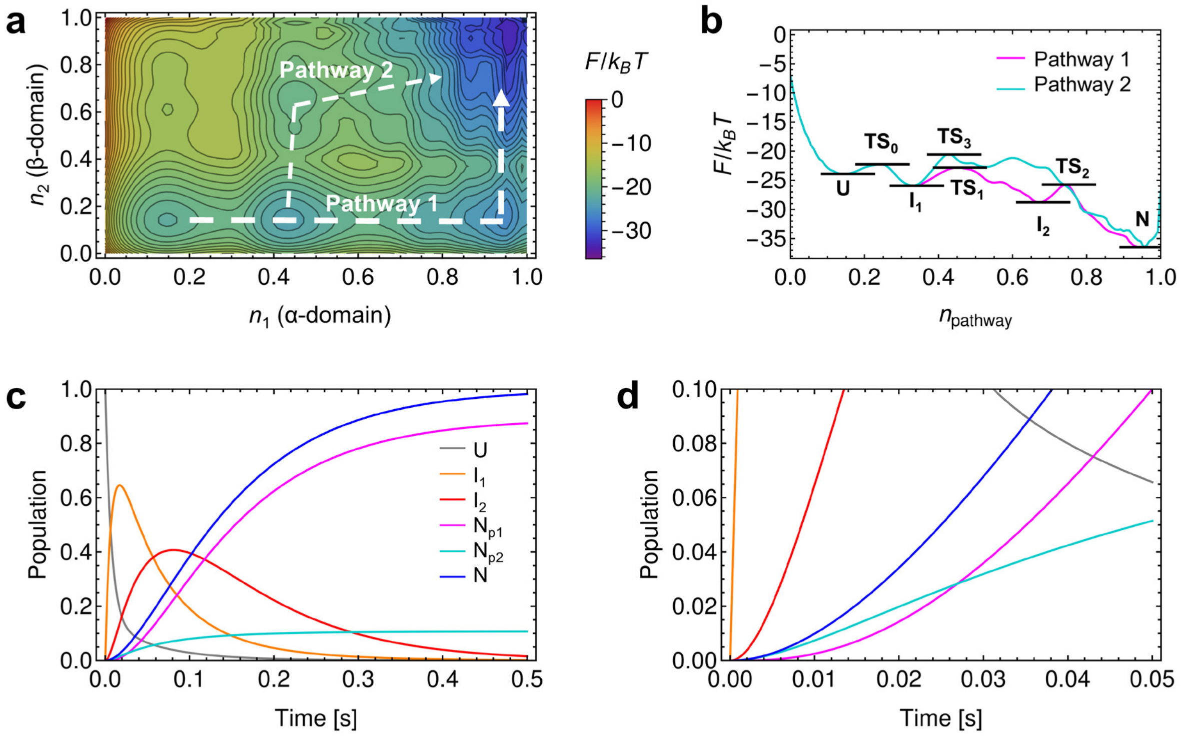

Supplementary Fig. 33. Kinetic analysis of lysozyme folding at 323 K.
a–d Details are shown in Supplementary Fig. 31. Source data are provided as a Source Data file.

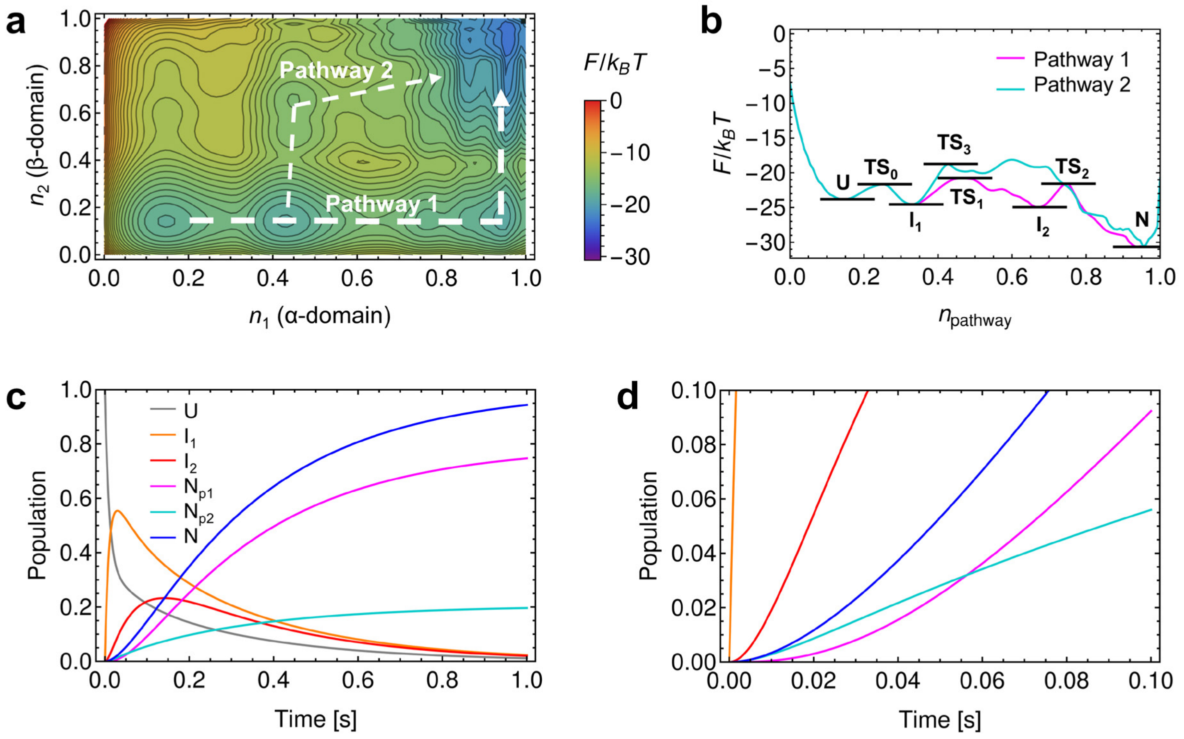

Supplementary Fig. 34. Kinetic analysis of lysozyme folding at 338 K.
a–d Details are shown in Supplementary Fig. 31. Source data are provided as a Source Data file.

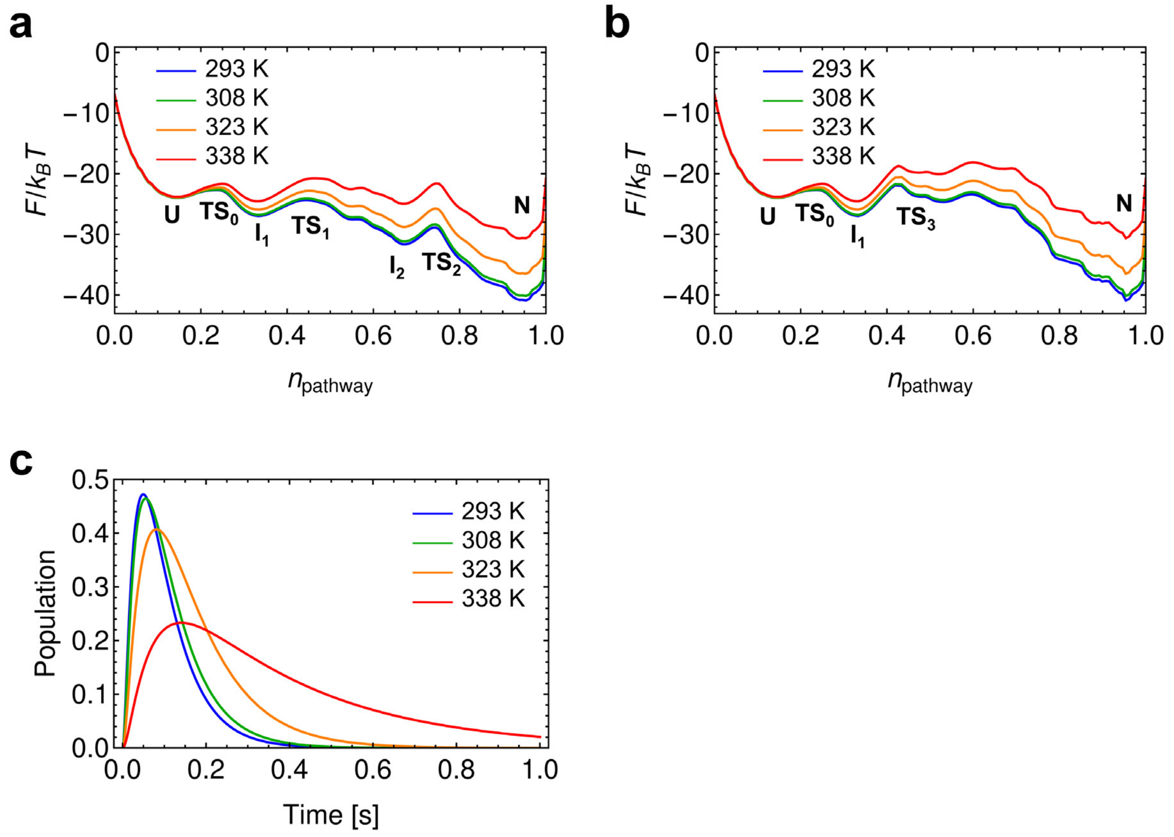

Supplementary Fig. 35. Temperature dependence of free energy landscape of lysozyme.
a, b Temperature dependence of cross-section of two-dimensional free energy landscape along Pathways 1 (**a**) and 2 (**b**) (Supplementary Figs. 31–34). **c** Population of I_2 intermediate during folding of lysozyme calculated at different temperatures. Source data are provided as a Source Data file.

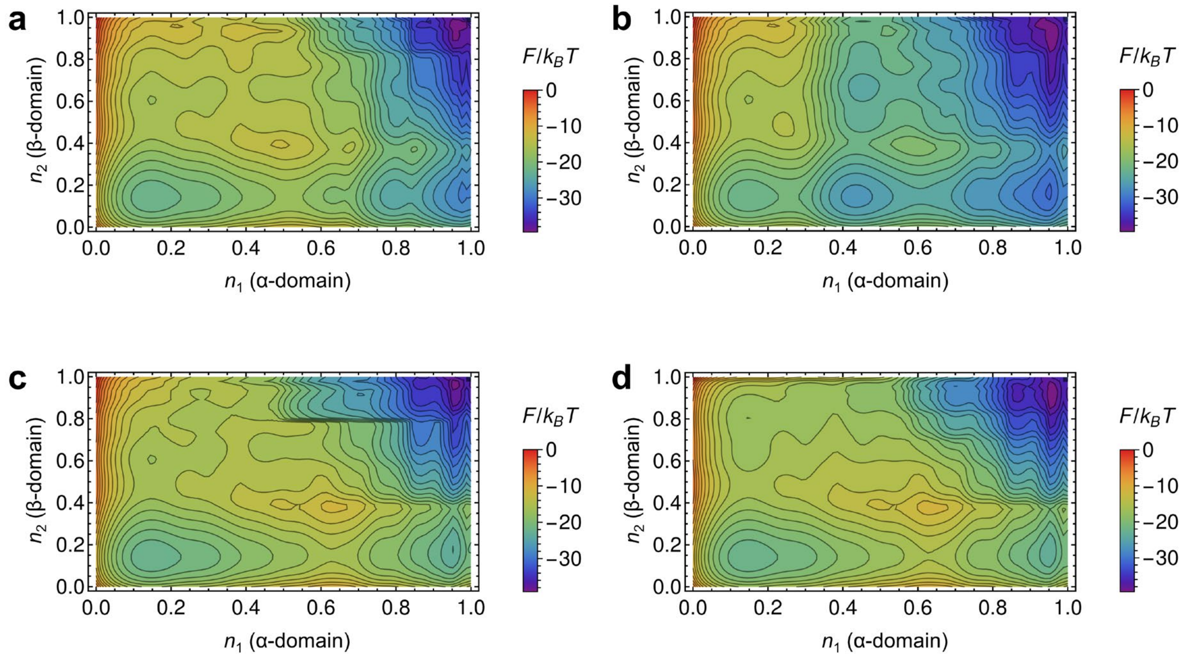

Supplementary Fig. 36. Two-dimensional free energy landscapes of lysozyme with a single disulfide bond calculated using the WSME-L(SS_{intact}) model.
Single disulfide bonds were introduced at 6–127 (**a**), 30–115 (**b**), 76–94 (**c**), and 64–80 (**d**). Source data are provided as a Source Data file.

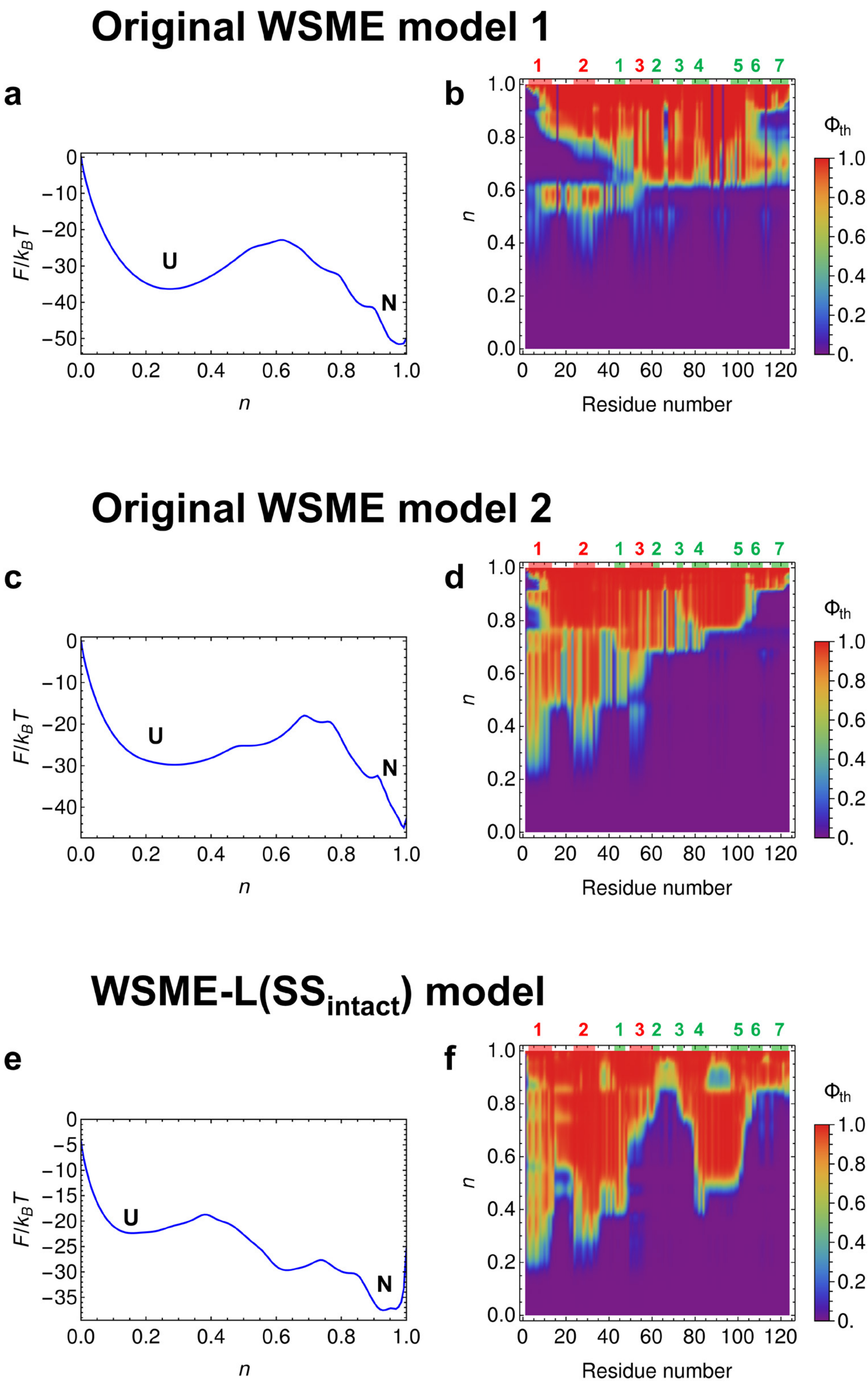

Supplementary Fig. 37. Folding of disulfide-intact ribonuclease A (RNase A). I.
a–f Details are shown in Supplementary Fig. 29. Source data are provided as a Source Data file.

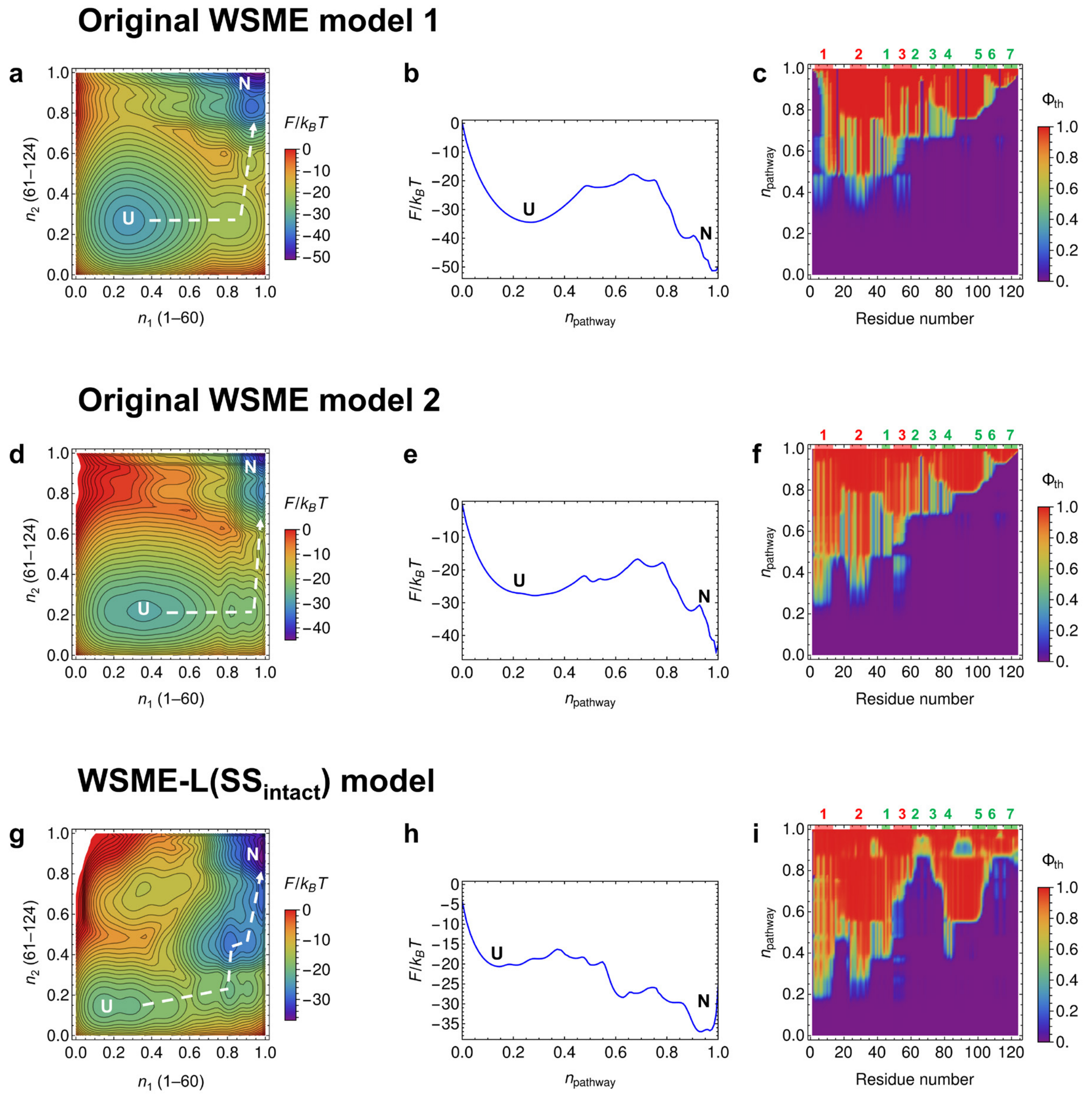

Supplementary Fig. 38. Folding of disulfide-intact ribonuclease A (RNase A). II.
a–i Details are shown in Supplementary Fig. 10. n_1 and n_2 are order parameters for magenta and cyan regions, respectively, as shown in Supplementary Fig. 24a. Source data are provided as a Source Data file.

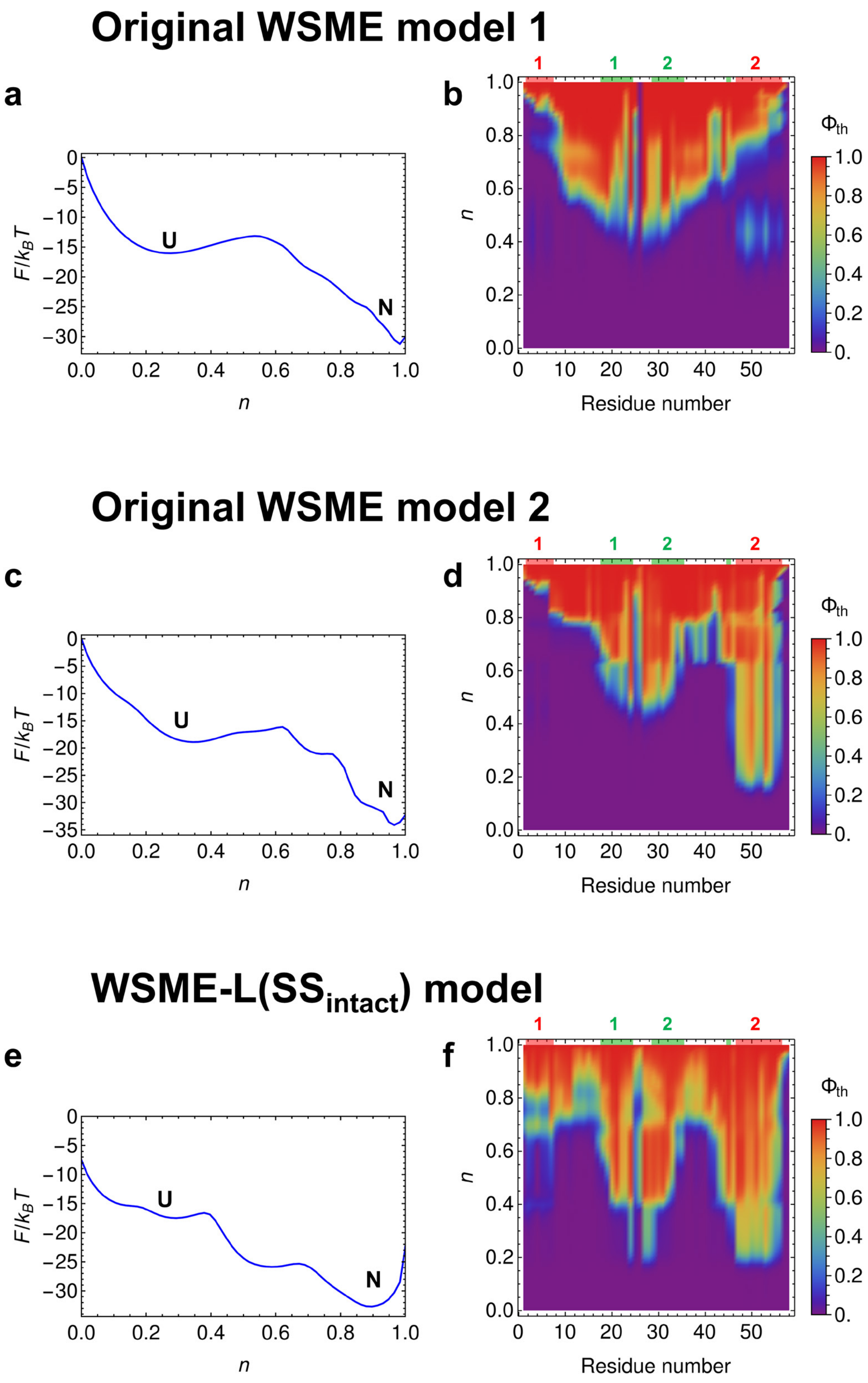

Supplementary Fig. 39. Folding of disulfide-intact bovine pancreatic trypsin inhibitor (BPTI). I.
a–f Details are shown in Supplementary Fig. 29. Source data are provided as a Source Data file.

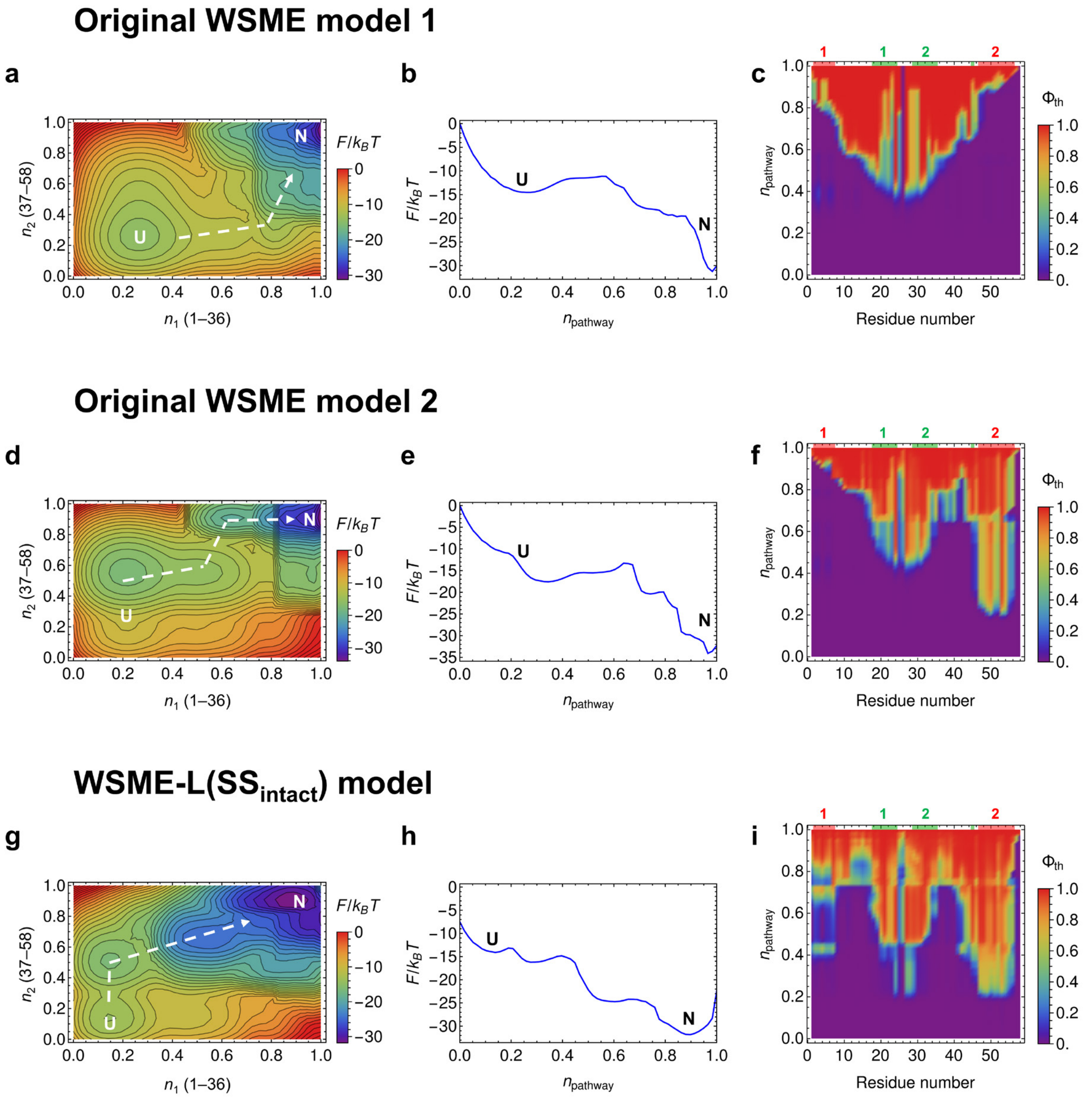

Supplementary Fig. 40. Folding of disulfide-intact bovine pancreatic trypsin inhibitor (BPTI). II.

a–i Details are shown in Supplementary Fig. 10. n_1 and n_2 are order parameters for magenta and cyan regions, respectively, as shown in Supplementary Fig. 26a. Source data are provided as a Source Data file.

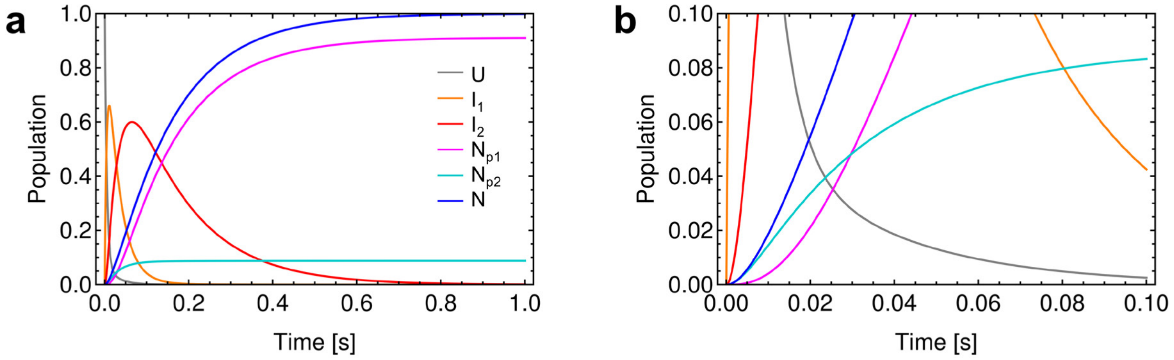

Supplementary Fig. 41. Kinetic analysis of lysozyme folding considering non-native interactions.

a Time evolution of concentrations of kinetic species calculated from two-dimensional free energy landscape at 293 K, assuming that non-native interactions stabilize I_2 intermediate by 0.44 kcal/mol. Total concentration was normalized to 1. **b** Expanded view of time evolution during first 0.1 s. Color scheme is same as that shown in **a**. Source data are provided as a Source Data file.

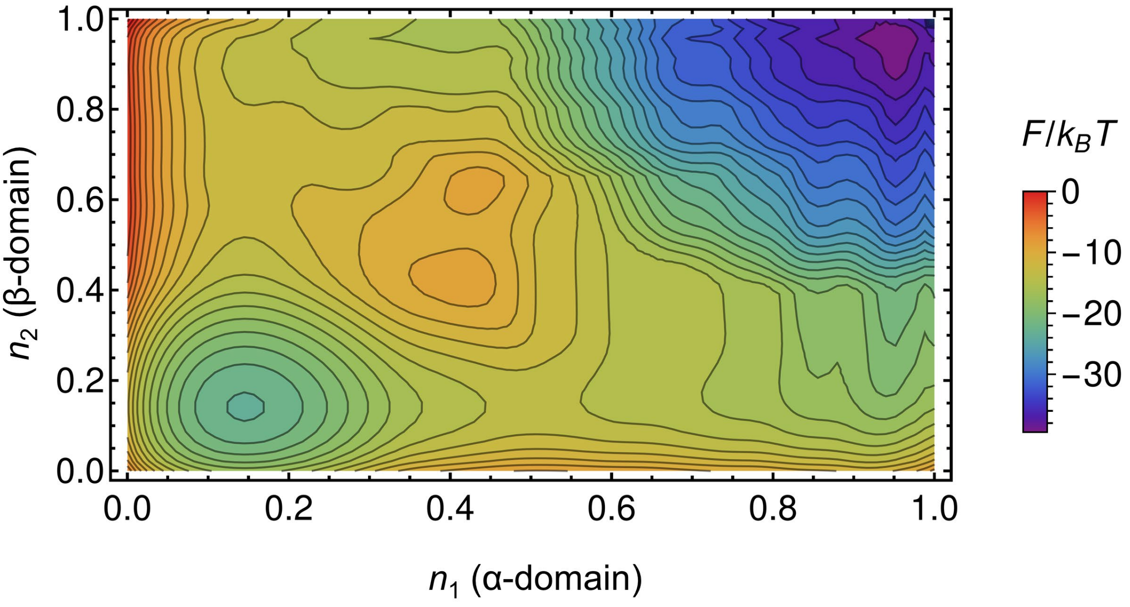

Supplementary Fig. 42. Two-dimensional free energy landscape of lysozyme calculated by WSME-L model using uniform contact energy at 293 K.
Uniform contact map for Original model 1 was used in this calculation. A uniform contact energy ε of 0.3817 was used. Source data are provided as a Source Data file.

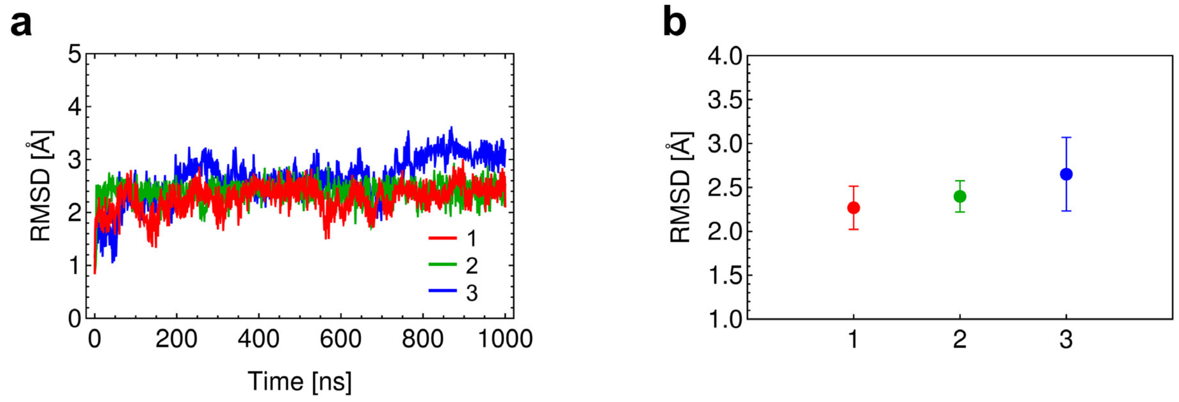

Supplementary Fig. 43. Molecular dynamics simulations of apomyoglobin.
a Trajectories of three independent 1-μs simulations after equilibration, represented by the root mean square deviation (RMSD) of the main-chain C_α atoms. The initial structure generated by the LEaP module was used as the reference structure. **b** Average and standard deviation of each trajectory in terms of RMSD. Source data are provided as a Source Data file.

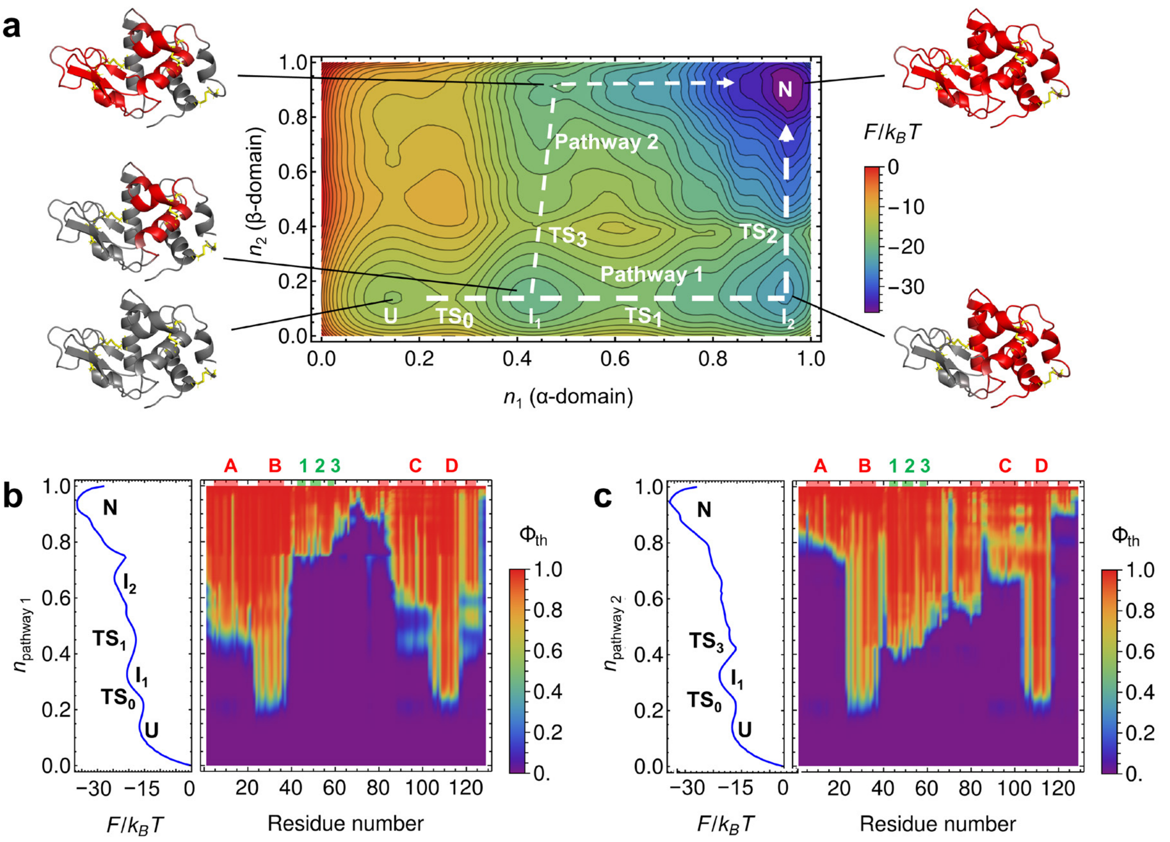

Supplementary Fig. 44. Folding of disulfide-intact lysozyme with simultaneous introduction of four linkers.

a Two-dimensional (2D) free energy landscape at 293 K. n_1 and n_2 are order parameters for α- and β-domains, respectively. White dashed lines indicate Pathways 1 and 2. Lysozyme residues predicted to be folded by theoretical Φ-value analysis are shown in red for structures of intermediate and native states. U and N denote unfolded and native states, respectively, I_1 and I_2 denote intermediates, and TS_0, TS_1, TS_2, and TS_3 denote transition states. **b**, **c** Residue-specific structure formation along folding pathways of lysozyme as predicted by theoretical Φ-value analysis. The Φ_{th}-values along Pathways 1 (**b**) and 2 (**c**) are plotted against residue number. Cross-section of 2D free energy landscape along folding pathway is shown on left. Red and green boxes on top frame indicate locations of helices and strands, respectively, and their names are shown in the corresponding colors. Source data are provided as a Source Data file.

Supplementary References

1. Gianni, S. *et al.* Unifying features in protein-folding mechanisms. *Proc. Natl. Acad. Sci. U.S.A.* **100**, 13286-13291 (2003).
2. Martinez, J. C. & Serrano, L. The folding transition state between SH3 domains is conformationally restricted and evolutionarily conserved. *Nat. Struct. Biol.* **6**, 1010-1016 (1999).
3. Riddle, D. S. *et al.* Experiment and theory highlight role of native state topology in SH3 folding. *Nat. Struct. Biol.* **6**, 1016-1024 (1999).
4. Garcia-Mira, M. M., Boehringer, D. & Schmid, F. X. The folding transition state of the cold shock protein is strongly polarized. *J. Mol. Biol.* **339**, 555-569 (2004).
5. Itzhaki, L. S., Otzen, D. E. & Fersht, A. R. The structure of the transition state for folding of chymotrypsin inhibitor 2 analysed by protein engineering methods: evidence for a nucleation-condensation mechanism for protein folding. *J. Mol. Biol.* **254**, 260-288 (1995).
6. Villegas, V., Martinez, J. C., Aviles, F. X. & Serrano, L. Structure of the transition state in the folding process of human procarboxypeptidase A2 activation domain. *J. Mol. Biol.* **283**, 1027-1036 (1998).
7. Eliezer, D. & Wright, P. E. Is apomyoglobin a molten globule? Structural characterization by NMR. *J. Mol. Biol.* **263**, 531-538 (1996).
8. Dyson, H. J. & Wright, P. E. How does your protein fold? Elucidating the apomyoglobin folding pathway. *Acc. Chem. Res.* **50**, 105-111 (2017).
9. Nishimura, C. Folding of apomyoglobin: Analysis of transient intermediate structure during refolding using quick hydrogen deuterium exchange and NMR. *Proc Jpn Acad Ser B Phys Biol Sci* **93**, 10-27 (2017).
10. Serrano, L., Matouschek, A. & Fersht, A. R. The folding of an enzyme. III. Structure of the transition state for unfolding of barnase analysed by a protein engineering procedure. *J. Mol. Biol.* **224**, 805-818 (1992).
11. Matouschek, A., Kellis, J. T., Jr., Serrano, L. & Fersht, A. R. Mapping the transition state and pathway of protein folding by protein engineering. *Nature* **340**, 122-126 (1989).
12. Matouschek, A., Kellis, J. T., Jr., Serrano, L., Bycroft, M. & Fersht, A. R. Transient folding intermediates characterized by protein engineering. *Nature* **346**, 440-445 (1990).
13. Fersht, A. *Structure and mechanism in protein science*. Vol. Volume 9 (WORLD SCIENTIFIC, 2017).
14. Raschke, T. M. & Marqusee, S. The kinetic folding intermediate of ribonuclease H resembles the acid molten globule and partially unfolded molecules detected under native conditions. *Nat Struct Biol* **4**, 298-304 (1997).
15. Raschke, T. M., Kho, J. & Marqusee, S. Confirmation of the hierarchical folding of RNase H: a protein engineering study. *Nat. Struct. Biol.* **6**, 825-831 (1999).
16. Hu, W. *et al.* Stepwise protein folding at near amino acid resolution by hydrogen exchange and mass spectrometry. *Proc. Natl. Acad. Sci. U.S.A.* **110**, 7684-7689 (2013).
17. Jennings, P. A., Finn, B. E., Jones, B. E. & Matthews, C. R. A reexamination of the folding mechanism of dihydrofolate reductase from *Escherichia coli*: verification and refinement of a four-channel model. *Biochemistry* **32**, 3783-3789 (1993).
18. Jones, B. E., Jennings, P. A., Pierre, R. A. & Matthews, C. R. Development of nonpolar surfaces in the folding of *Escherichia coli* dihydrofolate reductase detected by 1-anilinonaphthalene-8-sulfonate binding. *Biochemistry* **33**, 15250-15258 (1994).
19. Arai, M. *et al.* Microsecond hydrophobic collapse in the folding of *Escherichia coli* dihydrofolate reductase, an α/β-type protein. *J. Mol. Biol.* **368**, 219-229 (2007).
20. Arai, M., Iwakura, M., Matthews, C. R. & Bilsel, O. Microsecond subdomain folding in dihydrofolate reductase. *J. Mol. Biol.* **410**, 329-342 (2011).
21. Bilsel, O., Zitzewitz, J. A., Bowers, K. E. & Matthews, C. R. Folding mechanism of the

α-subunit of tryptophan synthase, an α/β barrel protein: global analysis highlights the interconversion of multiple native, intermediate, and unfolded forms through parallel channels. *Biochemistry* **38**, 1018-1029 (1999).
22. Wu, Y. & Matthews, C. R. Proline replacements and the simplification of the complex, parallel channel folding mechanism for the alpha subunit of Trp synthase, a TIM barrel protein. *J Mol Biol* **330**, 1131-1144 (2003).
23. Wu, Y., Vadrevu, R., Kathuria, S., Yang, X. & Matthews, C. R. A tightly packed hydrophobic cluster directs the formation of an off-pathway sub-millisecond folding intermediate in the α subunit of tryptophan synthase, a TIM barrel protein. *J Mol Biol* **366**, 1624-1638 (2007).
24. Gu, Z., Rao, M. K., Forsyth, W. R., Finke, J. M. & Matthews, C. R. Structural analysis of kinetic folding intermediates for a TIM barrel protein, indole-3-glycerol phosphate synthase, by hydrogen exchange mass spectrometry and Go model simulation. *J Mol Biol* **374**, 528-546 (2007).
25. Radford, S. E., Dobson, C. M. & Evans, P. A. The folding of hen lysozyme involves partially structured intermediates and multiple pathways. *Nature* **358**, 302-307 (1992).
26. Miranker, A., Robinson, C. V., Radford, S. E., Aplin, R. T. & Dobson, C. M. Detection of transient protein folding populations by mass spectrometry. *Science* **262**, 896-900 (1993).
27. Dobson, C. M., Evans, P. A. & Radford, S. E. Understanding how proteins fold: The lysozyme story so far. *Trends Biochem. Sci.* **19**, 31-37 (1994).
28. Kiefhaber, T. Kinetic traps in lysozyme folding. *Proc. Natl. Acad. Sci. U.S.A.* **92**, 9029-9033 (1995).
29. Radford, S. E. & Dobson, C. M. Insights into protein folding using physical techniques: Studies of lysozyme and α-lactalbumin. *Philos. Trans. R. Soc. Lond. B Biol. Sci.* **348**, 17-25 (1995).
30. Wildegger, G. & Kiefhaber, T. Three-state model for lysozyme folding: Triangular folding mechanism with an energetically trapped intermediate. *J. Mol. Biol.* **270**, 294-304 (1997).
31. Matagne, A., Radford, S. E. & Dobson, C. M. Fast and slow tracks in lysozyme folding: insight into the role of domains in the folding process. *J. Mol. Biol.* **267**, 1068-1074 (1997).
32. Dobson, C. M., Sali, A. & Karplus, M. Protein folding: A perspective from theory and experiment. *Angew. Chem. Int. Ed. Engl.* **37**, 868-893 (1998).
33. Matagne, A. *et al.* The origin of the α-domain intermediate in the folding of hen lysozyme. *J. Mol. Biol.* **277**, 997-1005 (1998).
34. Segel, D. J. *et al.* Characterization of transient intermediates in lysozyme folding with time-resolved small-angle X-ray scattering. *J. Mol. Biol.* **288**, 489-499 (1999).
35. van den Berg, B., Chung, E. W., Robinson, C. V., Mateo, P. L. & Dobson, C. M. The oxidative refolding of hen lysozyme and its catalysis by protein disulfide isomerase. *EMBO J* **18**, 4794-4803 (1999).
36. Matagne, A. *et al.* Thermal unfolding of an intermediate is associated with non-Arrhenius kinetics in the folding of hen lysozyme. *J. Mol. Biol.* **297**, 193-210 (2000).
37. Bieri, O. & Kiefhaber, T. Origin of apparent fast and non-exponential kinetics of lysozyme folding measured in pulsed hydrogen exchange experiments. *J. Mol. Biol.* **310**, 919-935 (2001).
38. Guez, V., Roux, P., Navon, A. & Goldberg, M. E. Role of individual disulfide bonds in hen lysozyme early folding steps. *Protein Sci.* **11**, 1136-1151 (2002).
39. Arai, K., Shibagaki, W., Shinozaki, R. & Iwaoka, M. Reinvestigation of the oxidative folding pathways of hen egg white lysozyme: switching of the major pathways by temperature control. *Int J Mol Sci* **14**, 13194-13212 (2013).

40. Udgaonkar, J. B. & Baldwin, R. L. NMR evidence for an early framework intermediate on the folding pathway of ribonuclease A. *Nature* **335**, 694-699 (1988).
41. Udgaonkar, J. B. & Baldwin, R. L. Early folding intermediate of ribonuclease A. *Proc. Natl. Acad. Sci. U.S.A.* **87**, 8197-8201 (1990).
42. Houry, W. A. & Scheraga, H. A. Structure of a hydrophobically collapsed intermediate on the conformational folding pathway of ribonuclease A probed by hydrogen-deuterium exchange. *Biochemistry* **35**, 11734-11746 (1996).
43. Houry, W. A., Rothwarf, D. M. & Scheraga, H. A. Circular dichroism evidence for the presence of burst-phase intermediates on the conformational folding pathway of ribonuclease A. *Biochemistry* **35**, 10125-10133 (1996).
44. Xu, X., Rothwarf, D. M. & Scheraga, H. A. Nonrandom distribution of the one-disulfide intermediates in the regeneration of ribonuclease A. *Biochemistry* **35**, 6406-6417 (1996).
45. Neira, J. L. & Rico, M. Folding studies on ribonuclease A, a model protein. *Fold Des* **2**, R1-11 (1997).
46. Rothwarf, D. M., Li, Y. J. & Scheraga, H. A. Regeneration of bovine pancreatic ribonuclease A: detailed kinetic analysis of two independent folding pathways. *Biochemistry* **37**, 3767-3776 (1998).
47. Wedemeyer, W. J., Welker, E., Narayan, M. & Scheraga, H. A. Disulfide bonds and protein folding. *Biochemistry* **39**, 4207-4216 (2000).
48. Shin, H. C., Song, M. C. & Scheraga, H. A. Effect of protein disulfide isomerase on the rate-determining steps of the folding of bovine pancreatic ribonuclease A. *FEBS Lett.* **521**, 77-80 (2002).
49. Arai, K., Kumakura, F. & Iwaoka, M. Kinetic and thermodynamic analysis of the conformational folding process of SS-reduced bovine pancreatic ribonuclease A using a selenoxide reagent with high oxidizing ability. *FEBS Open Bio* **2**, 60-70 (2012).
50. Weissman, J. S. & Kim, P. S. Reexamination of the folding of BPTI: predominance of native intermediates. *Science* **253**, 1386-1393 (1991).
51. Bulaj, G. & Goldenberg, D. P. Early events in the disulfide-coupled folding of BPTI. *Protein Sci.* **8**, 1825-1842 (1999).
52. Dadlez, M. & Kim, P. S. A third native one-disulphide intermediate in the folding of bovine pancreatic trypsin inhibitor. *Nat. Struct. Biol.* **2**, 674-679 (1995).
53. Kibria, F. M. & Lees, W. J. Balancing conformational and oxidative kinetic traps during the folding of bovine pancreatic trypsin inhibitor (BPTI) with glutathione and glutathione disulfide. *J. Am. Chem. Soc.* **130**, 796-797 (2008).
54. Andreeva, A., Kulesha, E., Gough, J. & Murzin, A. G. The SCOP database in 2020: expanded classification of representative family and superfamily domains of known protein structures. *Nucleic Acids Res.* **48**, D376-D382 (2020).
