# Supplementary material for: Accurate prediction of protein folding mechanisms by simple structure-based statistical mechanical models
Source: Nat Commun. 2023 Oct 19;14:6338. doi: 10.1038/s41467-023-41664-1 (PMC10587348; doi:10.1038/s41467-023-41664-1)
Supplement: Supplementary file 3 — Description of additional supplementary files [file 41467_2023_41664_MOESM3_ESM.pdf]

Description of Additional Supplementary Files Document

File Name: Supplementary Data 1

Description: The computational model structure of apomyoglobin in PDB format obtained from molecular dynamics simulations.
