# Supplementary material for: Accurate prediction of protein folding mechanisms by simple structure-based statistical mechanical models
Source: Nat Commun. 2023 Oct 19;14:6338. doi: 10.1038/s41467-023-41664-1 (PMC10587348; doi:10.1038/s41467-023-41664-1)
Supplement: Supplementary file 5 — Reporting Summary [file 41467_2023_41664_MOESM5_ESM.pdf]

nature portfolio

Corresponding author(s): Munehito Arai

Last updated by author(s): Aug 28, 2023

Reporting Summary

Nature Portfolio wishes to improve the reproducibility of the work that we publish. This form provides structure for consistency and transparency in reporting. For further information on Nature Portfolio policies, see our Editorial Policies and the Editorial Policy Checklist.

Statistics

For all statistical analyses, confirm that the following items are present in the figure legend, table legend, main text, or Methods section.

n/a	Confirmed	
☒	☐	The exact sample size (*n*) for each experimental group/condition, given as a discrete number and unit of measurement
☒	☐	A statement on whether measurements were taken from distinct samples or whether the same sample was measured repeatedly
☐	☒	The statistical test(s) used AND whether they are one- or two-sided<br>*Only common tests should be described solely by name; describe more complex techniques in the Methods section.*
☒	☐	A description of all covariates tested
☒	☐	A description of any assumptions or corrections, such as tests of normality and adjustment for multiple comparisons
☐	☒	A full description of the statistical parameters including central tendency (e.g. means) or other basic estimates (e.g. regression coefficient) AND variation (e.g. standard deviation) or associated estimates of uncertainty (e.g. confidence intervals)
☐	☒	For null hypothesis testing, the test statistic (e.g. *F*, *t*, *r*) with confidence intervals, effect sizes, degrees of freedom and *P* value noted<br>*Give P values as exact values whenever suitable.*
☒	☐	For Bayesian analysis, information on the choice of priors and Markov chain Monte Carlo settings
☒	☐	For hierarchical and complex designs, identification of the appropriate level for tests and full reporting of outcomes
☐	☒	Estimates of effect sizes (e.g. Cohen's *d*, Pearson's *r*), indicating how they were calculated

Our web collection on statistics for biologists contains articles on many of the points above.

Software and code

Policy information about availability of computer code

Data collection	AmberTools19 was used for energy minimization of protein structures and calculation of contact energies.<br>GROMACS 2021.2 and AmberTools19 were used for molecular dynamics (MD) simulations.<br>Custom codes written in C++ were used to generate folding free energy landscapes using the WSME-L, WSME-L(SS), and WSME-L(SSintact) models. The codes are available at https://github.com/ut-arailab/WSME-L_model, which is archived in Zenodo with the identifier [https://doi.org/10.5281/zenodo.8280372].
Data analysis	Mathematica 12.2.0.0 was used to draw contact maps and free energy landscapes.<br>AmberTools19 was used for analysis of MD simulations.

For manuscripts utilizing custom algorithms or software that are central to the research but not yet described in published literature, software must be made available to editors and reviewers. We strongly encourage code deposition in a community repository (e.g. GitHub). See the Nature Portfolio guidelines for submitting code & software for further information.

Data

Policy information about availability of data

All manuscripts must include a data availability statement. This statement should provide the following information, where applicable:

- Accession codes, unique identifiers, or web links for publicly available datasets
- A description of any restrictions on data availability
- For clinical datasets or third party data, please ensure that the statement adheres to our policy

The protein structures used in this study are available in PDB under accession codes: 2jwt [https://doi.org/10.2210/pdb2jwt/pdb] (En-HD), 4jz4 [https://doi.org/10.2210/pdb4jz4/pdb] (src SH3), 1u06 [https://doi.org/10.2210/pdb1u06/pdb] (α-spectrin SH3), 1csp [https://doi.org/10.2210/pdb1csp/pdb] (CspB), 7a1h

[https://doi.org/10.2210/pdb7a1h/pdb] (CI2), 1aye [https://doi.org/10.2210/pdb1aye/pdb] (ADA2h), 1bzp [https://doi.org/10.2210/pdb1bzp/pdb] (apoMb), 1a2p [https://doi.org/10.2210/pdb1a2p/pdb] (barnase), 7vsc [https://doi.org/10.2210/pdb7vsc/pdb] (RNase HI), 5uih [https://doi.org/10.2210/pdb5uih/pdb] (DHFR), 1jul [https://doi.org/10.2210/pdb1jul/pdb] (IGPS), 1iee [https://doi.org/10.2210/pdb1iee/pdb] (lysozyme), 6etl [https://doi.org/10.2210/pdb6etl/pdb] (RNase A) , and 5pti [https://doi.org/10.2210/pdb5pti/pdb] (BPTI) and in AlphaFold Protein Structure Database under accession code: P0A877 [https://alphafold.ebi.ac.uk/entry/P0A877] (αTS). The computational model structure of apoMb generated in this study is provided in the Supplementary Data file. All data generated or analyzed during this study are included in this article and its supplementary information files. Source data are provided with this paper.

Field-specific reporting

Please select the one below that is the best fit for your research. If you are not sure, read the appropriate sections before making your selection.

☒ Life sciences ☐ Behavioural & social sciences ☐ Ecological, evolutionary & environmental sciences

For a reference copy of the document with all sections, see nature.com/documents/nr-reporting-summary-flat.pdf

Life sciences study design

All studies must disclose on these points even when the disclosure is negative.

Sample size	To demonstrate the general applicability of our WSME-L models to a variety of proteins, we selected representative proteins whose folding reaction mechanisms have been studied in detail, covering a wide range of structural classes and numbers of amino acid residues. One-microsecond MD simulations for apoMb were performed in triplicate. The number of replicates and their timescales were sufficient to obtain the computational model structure of apoMb that provides the free energy landscape consistent with experiment.
Data exclusions	No data were excluded from the analyses.
Replication	One-microsecond MD simulations for apoMb were performed in triplicate.
Randomization	Randomization was not performed because the data were collected without selection. No animals or human subjects were involved in this study.
Blinding	This item is not relevant because the results were quantitative and did not require subjective judgment or interpretation. No animals or human subjects were involved in this study.

Reporting for specific materials, systems and methods

We require information from authors about some types of materials, experimental systems and methods used in many studies. Here, indicate whether each material, system or method listed is relevant to your study. If you are not sure if a list item applies to your research, read the appropriate section before selecting a response.

Materials & experimental systems

n/a	Involved in the study	
☒	☐	Antibodies
☒	☐	Eukaryotic cell lines
☒	☐	Palaeontology and archaeology
☒	☐	Animals and other organisms
☒	☐	Human research participants
☒	☐	Clinical data
☒	☐	Dual use research of concern

Methods

n/a	Involved in the study	
☒	☐	ChIP-seq
☒	☐	Flow cytometry
☒	☐	MRI-based neuroimaging
